# Extracellular vesicles from biological fluids as potential markers in castration resistant prostate cancer

**DOI:** 10.1007/s00432-022-04391-6

**Published:** 2022-10-12

**Authors:** Wendy W. Y. Choi, Catherine Sánchez, Jiao Jiao Li, Mojdeh Dinarvand, Hans Adomat, Mazyar Ghaffari, Leila Khoja, Fatemeh Vafaee, Anthony M. Joshua, Kim N. Chi, Emma S. Tomlinson Guns, Elham Hosseini-Beheshti

**Affiliations:** 1 Vancouver Prostate Centre, 2660 Oak St, Vancouver, BC V6H 3Z6 Canada; 2 Las Condes Clinic, Estoril 450, Las Condes, Santiago, Chile; 3 Kolling Institute, Faculty of Medicine and Health, The University of Sydney, St Leonards, NSW 2065 Australia; 4 School of Biomedical Engineering, Faculty of Engineering and IT, University of Technology Sydney, Ultimo, NSW 2007 Australia; 5 School of Biotechnology and Biomolecular Sciences, University of New South Wales, Kensington, NSW 2052 Australia; 6 Sydney Medical School, Faculty of Medicine and Health, The University of Sydney, Sydney, NSW 2006 Australia; 7 St Vincent’s Hospital Sydney, Darlinghurst, NSW 2010 Australia; 8 UNSW Data Science Hub, University of New South Wales, Kensington, NSW 2052 Australia; 9 BC Cancer Agency, 600 West 10th Avenue, Vancouver, BC V5Z 4E6 Canada; 10 School of Medical Sciences, Faculty of Medicine and Health, The University of Sydney, Sydney, NSW 2006 Australia; 11 The Sydney Nano Institute, The University of Sydney, Sydney, NSW 2006 Australia

**Keywords:** Prostate cancer, Extracellular vesicles, Proteomic, Cholesterol, Liquid biopsy, Cancer markers

## Abstract

**Purpose:**

Extracellular vesicles (EV) secreted from cancer cells are present in various biological fluids, carrying distinctly different cellular components compared to normal cells, and have great potential to be used as markers for disease initiation, progression, and response to treatment. This under-utilised tool provides insights into a better understanding of prostate cancer.

**Methods:**

EV from serum and urine of healthy men and castration-resistant prostate cancer (CRPC) patients were isolated and characterised by transmission electron microscopy, particle size analysis, and western blot. Proteomic and cholesterol liquid chromatography-mass spectrometry (LC–MS) analyses were conducted.

**Results:**

There was a successful enrichment of small EV/exosomes isolated from serum and urine. EV derived from biological fluids of CRPC patients had significant differences in composition when compared with those from healthy controls. Analysis of matched serum and urine samples from six prostate cancer patients revealed specific EV proteins common in both types of biological fluid for each patient.

**Conclusion:**

Some of the EV proteins identified from our analyses have potential to be used as CRPC markers. These markers may depict a pattern in cancer progression through non-invasive sample collection.

**Supplementary Information:**

The online version contains supplementary material available at 10.1007/s00432-022-04391-6.

## Introduction

Biological fluids such as blood and urine are ideal sources for prostate cancer (PCa) marker detection since they can be easily obtained and avoid the need for invasive prostate biopsies. After the successful completion of the Human Genome Project, and with the support of the Human Proteome Organisation, there has been increasing global effect on mapping the human proteome and on using proteomic tools in biomarker research. Proteomic analysis of biological fluids has the potential to provide an overview of protein content in different organs and presents new opportunities for biomarker discovery in relation to cancer development and progression (Honda et al. 2013). Identifying proteins and their associated post-translational modifications in biological fluids such as blood and urine can be performed through a non-invasive procedure, which captures the entire heterogeneity of cancer progression through circulating biomarkers. Urine was suggested as an ideal biofluid for the diagnosis of urologic cancers such as PCa since it contains proteins originating from the bladder, prostate and kidney that could reflect physiological and pathological changes indicative of disease progression (Wood et al. 2013). However, the discovery of new PCa biomarkers from biological fluids has been hampered by the complexity of these fluids, especially since the molecules of interest are usually present in very low quantities.

Over the past decade, extracellular vesicles (EV) have been increasingly identified and studied for their essential roles in cellular communication as biological messengers for physiological (Doyle and Wang 2019; Yáñez-Mó et al. 2015) and pathological (Braicu et al. 2015; Zocco et al. 2014) processes. EV are considered a heterogeneous mixture of nanoscale vesicles with a size range of 50 nm to 5 µm, with exosomes, microvesicles, and apoptotic vesicles being the three major types of EV that have been intensely studied (Colombo et al. 2014). In addition, oncosomes are large cancer-derived EV (> 1 μm) that are associated with advanced disease and are being increasingly studied in recent years (Meehan et al. 2016; Minciacchi et al. 2015; Vagner et al. 2018). EV carry several classes of proteins (Wu et al. 2019) and genetic materials (DNA and RNA) (Huang et al. 2013; Thakur et al. 2014) and as such are a major potential source of diagnostic and prognostic markers. The accessibility of these vesicles in biological fluids such as blood (Caby et al. 2005), urine (Pisitkun et al. 2004), breast milk (Admyre et al. 2007)(Andersen, Berglund et al. 1997), saliva (Palanisamy et al. 2010), and bronchoalveolar lavage fluid (Admyre et al. 2003) has resulted in recent studies to exploit them as sources of biomarkers for various pathologies, including PCa (Minciacchi et al. 2017; Saber et al. 2020; Oey et al. 2021). Several in vitro studies have demonstrated that, compared to normal cells, cancer cells not only release a significantly higher number of EV (Ostrowski et al. 2010; Yu et al. 2006), but the protein and genetic content of these EV are also different (Bryzgunova et al. 2016; Cheng et al. 2020; Warnecke-Eberz et al. 2015). Our own study using five PCa cell lines has also clearly demonstrated significant differences in the proteomic and lipidomic content of PCa exosomes compared to benign epithelial prostate cell-derived exosomes (Hosseini-Beheshti et al. 2012).

Building on our previous findings, this study uses mass spectrometric (MS)-based proteomic and cholesterol profiling to compare the EV derived from biological fluids (blood and urine) between PCa patients and healthy individuals, specifically in a cohort of castration-resistant prostate cancer (CRPC) patients whose disease progression continues despite androgen depletion therapy. Although some previous studies have reported proteomic analyses on EV from PCa samples, these were performed using PCa cell lines (Minciacchi et al. 2015; Duijvesz et al. 2013) or urine samples only (Mitchell et al. 2009; Nilsson et al. 2009). This is the first study to perform EV analysis for PCa, 1) using both blood and urine samples of CRPC patients, including matched samples from 6 individuals and 2) using both proteomic and cholesterol profiling. EV derived from the biological fluids of CRPC patients had significantly different composition compared to those from healthy individuals. These findings are potentially useful for the identification of new indicators of PCa progression.

## Experimental procedures

### Patients

Fifteen CRPC patients and three healthy volunteers were included in this study. All CRPC patients were confirmed positive for PCa through prostate biopsy. Patient information including serum prostate-specific antigen (PSA) levels, age, and treatment history were collected as part of a clinical trial designed to study Abiraterone (clinical trials.gov number NCT01857908) and summarised in Table S1. The average age was 71 years (range 57–81) for CRPC patients (9 urine donors and 12 blood donors), and 29 years (range 26–33) for healthy control donors. The lower age range of the control donors was to ensure that these individuals had minimal chance for presenting with PCa or benign prostate hyperplasia. Six of the CRPC patients provided matched urine and blood samples for this study.

### Biological fluid samples

Biological fluid samples were obtained from all individuals included in this study. For the 15 CRPC patients, the samples were obtained as part of a baseline collection prior to commencing Abiraterone treatment. Whole blood to the volume of 5 mL was collected from 15 CRPC patients and 3 healthy volunteers by venipuncture in red-top, no additive tubes. The serum fraction of blood samples was used for analysis as our preliminary work indicated that the serum fraction yielded a higher number of identified proteins than plasma (Data not shown). Serum fractions were separated by centrifugation at 1000* g* for 20 min and frozen at -80 °C until further analysis. Urine samples were collected from 12 CRPC patients and 3 healthy volunteers, who provided a pooled 24-h urine collection of which 50 mL per patient was retained in a sterile container and frozen at − 80 °C until further analysis. All samples were collected and handled in accordance with the Human Ethics Board Approval Cert. H09-01,010 obtained from the University of British Columbia, Canada.

### EV isolation

EV were isolated using different methods for serum and urine samples. Serum samples were thawed and centrifuged at 500* g* for 5 min at 10 °C to remove any cell debris. The supernatant was transferred to a fresh centrifuge tube for the second centrifugation cycle at 3,000* g* for 20 min at 10 °C. Following this, the supernatant was again transferred to a fresh centrifuge tube for the third centrifugation cycle at 12,000* g* for 20 min at 10 °C. Ultracentrifugation was then performed on a 30% sucrose cushion at 100,000* g* for 70 min at 10 °C, using a fixed angle 70.1 Ti rotor (Beckman Coulter, USA). The resulting serum EV pellets (300 µL) were washed with PBS, followed by a final round of ultracentrifugation at 100,000* g* for 70 min at 10 °C. The EV pellets were washed and stored at -80 °C until further analysis.

Urine samples were thawed at room temperature, following which 2 tablets of protease inhibitors (cOmplete™, Roche Applied Science) were immediately added to a 25 mL volume of each sample. The samples were vortexed for 1 min and centrifuged at 500* g* for 5 min at 4 °C to remove cell debris. The cell-free urine supernatant was centrifuged at 17,000* g* for 20 min at 4 °C. The supernatant was recovered, and ultracentrifugation was performed on a 30% sucrose cushion at 200,000* g* for 70 min at 4 °C using a fixed angle 70.1 Ti rotor (Beckman Coulter). The resulting urine EV pellets (300 µL) were washed with PBS, followed by a final round of ultracentrifugation at 200,000* g* for 70 min at 4 °C. The EV pellets were washed and stored at -80 °C until further analysis.

### Transmission electron microscopy (TEM)

Isolated EV (2.5 μL) were dried onto freshly glow-discharged 300 mesh Formvar/carbon-coated TEM grids (Ted Pella, Redding, CA, USA) and negatively stained with 2% aqueous uracyl acetate. EV samples were visualised by TEM using Hitachi H7600 (Hitachi High-Technologies Corp., Tokyo, Japan) operated at 80 kV. Images were captured with a side mounted 1 K AMT Advantage digital camera (Advanced Microscopy Techniques, Corp. Woburn, MA, USA).

### Western blot

Total protein extract from serum, urine, and their isolated EVs were obtained by sonication and analysed for total protein concentration using the Pierce™ BCA protein assay kit (Thermo Fisher Scientific, USA). Total protein (30 mg) from each sample was loaded onto 12% acrylamide gels. Detection of exosome markers was conducted by western blot using the following antibodies: mouse monoclonal Alix, mouse monoclonal CD63, mouse monoclonal HSP70 and goat polyclonal HSP90 α/β (all 1:1000, Santa Cruz Biotechnology, USA); mouse monoclonal TSG101 (1:1000, Abnova, Taiwan); and rabbit polyclonal LAMP2 (1:1000, Abcam, UK).

### Nanoparticle tracking analysis (NTA)

Isolated EV were analysed for size distribution and the estimated concentration of nanoparticles using the NanoSight™ LM10 system (NanoSight Ltd, UK), configured with a 488-nm laser and a high-sensitivity digital camera (OrcaFlash 2.8, Hamamatsu C11440, NanoSight Ltd). This system analyses the EV using a light-scattering technology by measuring the rate of Brownian motion. Briefly, EV samples were diluted with nanoparticle-free water to achieve a concentration range of 5 × 10^7^ to 5 × 10^9^ nanoparticles/mL. Samples were infused and recorded under a controlled flow (infusion rate of 100) using the NanoSight™ syringe pump and script control system. The ambient temperature was set at 25 °C, camera sensitivity was set between 9 and 12, and detection threshold was set between 3 and 5 for optimal particle detection. Five different videos of 60 s from 3 different replicates for each sample were collected and analysed using NTA software (version 2.3).

### Proteomic analysis

An in-solution trypsin digestion protocol was used to generate peptides for liquid chromatography-mass spectrometry (LC–MS) analysis. Briefly, EV isolated from serum and urine were sonicated for 5 min, and a sample amount equivalent to 40 μg protein was precipitated with 5–10 × volumes of acetone at -20 °C for 1 h. The precipitate was centrifuged at 20,000* g* for 5 min, and the pellet was re-dissolved in 36 μL of 25 mM ammonium bicarbonate. Each sample was added with 1 μL of 100 mM dithiothreitol (DTT) solution and incubated for 35 min at 65 °C, followed by adding 2 μL of 100 mM iodoacetamide and incubating for an additional 30 min in the dark at room temperature. The sample was then added with 1 μL of 100 ng/µL trypsin and incubated overnight at 37 °C. The resulting peptides were passed through a 75 μm × 100 mm 1.7 μm BEH130 C18 column using a 3–40% linear acetonitrile gradient with 0.1% formic acid present throughout, at 0.3 μL/min over 40 min using a NanoAcquity™ LC (Waters, USA). The column was re-equilibrated for 20 min between runs. Column eluate was directed into a Synapt™ mass spectrometer through a 20 µm capillary held at 3.2 kV. Instrument calibration was performed using Glu-Fibrinogen fragments, and Glu-Fibrinogen was also used as a lock mass to compensate for any calibration drift. The instrument was run in V-mode with a mass resolution of approximately 10,000. A data-dependent method was used with a 1 s scan, followed by up to 3 fragment scans, using ion intensity and charge state as the main selection criteria. The accumulated data was analysed using ProteinLynx Global Server (PLGS) software with peptide and fragment mass accuracies of 25 ppm and 0.1 Da, respectively. Uniform carbamido-methyl C and variable N-terminal acetyl, M oxidation, N deamidation, and C propionamide were selected as permitted modifications with a maximum protein molecular weight of 250 K. This search engine was applied to the full Uniprot database, human species. A search with similar parameters was also performed in Mascot using the pkl peak list files generated in PLGS.

#### Gene ontology and pathway analyses

Enrichment analysis (Boyle et al. 2004) was used to determine whether known biological processes or pathways are over-represented by a list of proteins of interest. The enrichment p-value was estimated by Fisher’s exact test with hypergeometric null distribution, and p-values were adjusted for multiple hypothesis tests using false discovery rate (FDR) correction (Benjamini and Hochberg 1995). Gene ontology (GO) and pathway enrichment analyses were performed. Kyoto Encyclopedia of Genes and Genomes (KEGG) was used to retrieve pathway annotations, as well as the clusterProfiler package (Wu et al. 2019) which implements *enrichKEGG* function for pathway over-representation test. Adjusted p-value cut-off of 0.05 was used to identify significantly enriched terms. Protein UniProt IDs were converted to gene symbols using org.Hs.eg.db in R Bioconductor.

Similarly, GO enrichment test was performed using *enrichGO* function implemented by clusterProfiler package in R. GO comprises three orthogonal ontologies: cellular component (CC), biological process (BP), and molecular function (MF). GO terms are organised in a directed acyclic graph, where edges between terms represent parent–child relationship. Such relationships enable summarisation of related and redundant enriched GO terms (adjusted *p* value < 0.05) based on their semantic similarities, enhancing visualisation and interpretation of results. Pairwise semantic similarities between enriched GO terms were estimated using GOSemSim R package (Yu 2020) (distance method parameter was set to ‘Rel’). Then, the rrvgo package (Sayols and rrvgo, 2020) was used for plotting and interpreting the results. Scatter plot was used for depicting groups and distance between GO terms, where distance between points represented the similarity between terms, and axes were the first two principal components of applying a PCA to the similarity matrix. Size of the point represented the provided scores or, in its absence, the number of genes the GO term contained. Treemaps visualisation was used where terms were grouped based on their parent, and the space used by the term was proportional to the score. Treemaps can help with interpreting the summarised results and comparing different sets of GO terms (Sayols and rrvgo, 2020). Pie charts were also used for visualisation of GO terms, where the slice size was proportional to the level of significance of the corresponding term, such that a larger slice would represent a lower p-value. All R codes are available at: https://github.com/VafaeeLab/PCa_proteomics_analysis.

### Cholesterol analysis

Serum, urine, and their isolated EV were extracted (Matyash et al. 2008) and derivatised prior to analysis (Liebisch et al. 2006) according to published methods. Briefly, samples (5 µL serum, 20 µL serum-derived EV, or 100 µL urine/urine-derived EV) were spiked with 200 ng of deuterated cholesterol and vortexed with 1 mL of 20/80 MeOH/MTBE (methanol/methyl-t-butyl ether) for 30 min in glass tubes. The sample was then added with 500 μL of ddH_2_O, vortexed for another 10 min, and centrifuged for 5 min (Centrivap), after which the upper layer was collected. A second extraction using 1 mL of MeOH:MTBE was performed and pooled with the first, after which the extracts were dried in the Centrivap under vacuum. Dried extracts were dissolved in 200 μL of 1:5 acetyl chloride/chloroform solution and incubated for 1 h at room temperature. Samples were then left uncapped in a fume hood for 15 min before final drying in the Centrivap. Residue was re-dissolved in 60 µL of 70/30 methanol/chloroform, further diluted with 140 µL of methanol, and centrifuged at 15,000* g* for 5 min before transferring to LC vials.

LC–MS analysis was performed with Waters Acquity ultra performance liquid chromatography (UPLC) coupled to a Quattro Premier XE using a 2.1 × 50 mm BEH 1.7 µM C18 column. The mobile phase consisted of (A) 9/1 acetonitrile/0.1 M ammonium acetate and (B) isopropanol with the following gradient: 0.2 min, 25% B; 5–8 min, 70% B, 8.1 min, 25% B with a 10 min run length. Instrument parameters were optimised for the m/z of ammonium adducts of acetate derivatised cholesterol, and the m/z369 fragment was used for multiple reaction monitoring (MRM) quantitation. The area under curve (AUC) for cholesterol acetate and d6 cholesterol acetate were obtained using Quanlynx, and a linear calibration curve from 0.2–10 µg/mL, R^2^ > 0.99 was generated using AUC ratios.

### Statistical analysis

All data were expressed as mean ± standard deviation. Differences between groups were evaluated using the Student’s *t*-test, and *p* < 0.05 was considered statistically significant.

## Results

### Characterisation of EV derived from serum and urine

The presence of EV in serum and urine samples of CRPC patients and healthy individuals were confirmed using TEM, western blot, and NTA. Isolated small EV were visualised by TEM, which revealed the presence of cup-shaped nanovesicles with a size range of 30–200 nm for both serum (Figure S1A) and urine (Figure S1D) samples of CRPC patients and healthy controls.

Western blot was performed to confirm the presence of EV markers. The serum-derived EV contained LAMP2, TSG101 and CD63, which were distinct protein markers for exosomes (Figure S1B). Similarly, the urine-derived EV contained the EV markers Alix, HSP70, HSP 90, LAMP2 and TSG101, which were all enriched compared to the unprocessed urine samples (Figure S1E).

NTA was used to measure the size and concentration of EV and validate the EV isolation efficacy from biological fluid samples. For EV isolated from serum (Figure S1C) and urine (Figure S1F), 84–87% of the nanoparticles were within a size range of 30–200 nm (coinciding with exosomes/small EV), while 13–16% were within a size range of 200–1000 nm (coinciding with microvesicles/large EV). In the exosomal size range, the nanoparticle concentration of serum-derived EV was similar to that of unprocessed serum (data not shown), potentially due to the presence of serum proteins such as albumin and globulin as well as non-EV lipid particles such as chylomicrons and lipoprotein particles. The nanoparticle concentration of urine-derived EV was approximately two times higher than that of unprocessed urine (Figure S1G).

### Comparison of EV derived from serum and urine between PCa patients and controls

**Fig. 1:**
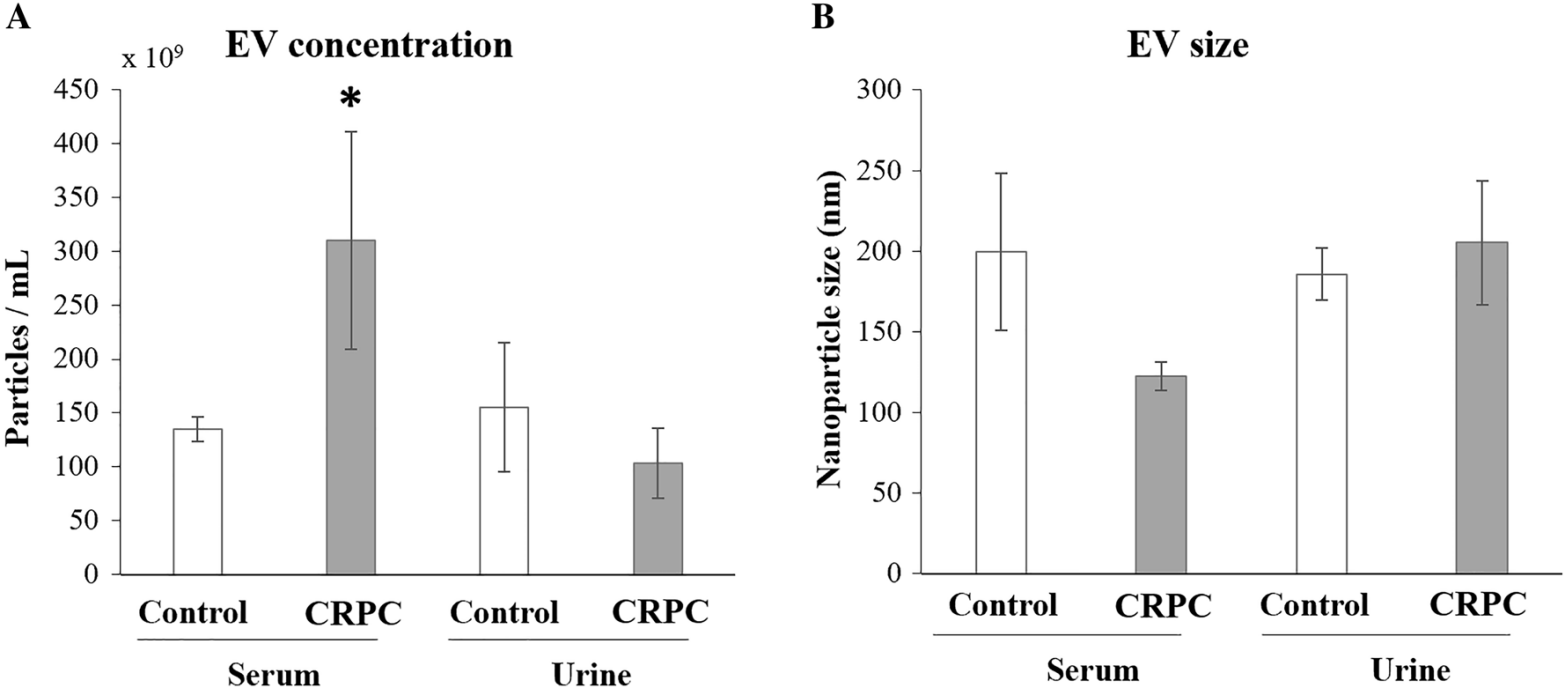
Nanoparticle tracking analysis for serum- and urine-derived EV. **A** Number of nanoparticles/mL and **B** average nanoparticle size (nm) after EV isolation. Serum samples: control *n* = 3, CRPC *n* = 15; urine samples: control *n* = 3, PCa *n* = 12. *p < 0.05

NTA results were used to compare the number and size of EV isolated from serum and urine between CRPC patients and healthy controls. For serum-derived EV, the number of nanoparticles (Fig. 1A) was significantly higher in CRPC patients compared to controls, while the size range (Fig. 1B) of nanoparticles from CRPC patients (104–159 nm) was slightly smaller than that from the control group (159–187 nm). For urine-derived EV, there were no significant differences in nanoparticle number or size range between CRPC patients and controls. In CRPC patients, EV derived from urine appeared to be less abundant with larger sizes compared to those derived from serum.

### Proteomic analysis of EV derived from serum and urine

#### Protein distribution in serum and serum-derived EV

The protein distribution and profile content of serum and serum-derived EV were compared by protein electrophoresis and Coomassie blue staining. The pattern of proteins was similar between unprocessed serum and serum-derived EV, but albumin level in the latter was significantly lower (Figure S2A). To decrease the albumin content of serum-derived EV and minimise their interference in subsequent proteomic MS analysis, samples were pre-cleared with AlbuminOUT™ columns (G-Biosciences, USA). This treatment not only 47decreased albumin content, but also reduced the amount of protein available in serum-derived EV samples for further analysis (Figure S2B).

#### LC–MS analysis of proteins from serum- and urine-derived EV

LC–MS was used to describe the protein profile of EV derived from CRPC patient serum (*n* = 12) and urine (*n* = 9) samples, with 2 biological replicates from each donor. IPA core and biomarker analyses were used for a rapid assessment of the signalling and metabolic pathways involved, and for identification of the most promising and relevant prostate-related candidates (supplementary information). These analyses identified 159 serum-derived and 78 urine-derived EV proteins in CRPC patient samples. Overall, 182 and 85 EV proteins were identified from all CRPC and control samples of serum and urine, respectively. A list of identified proteins with their protein ID, subcellular location, type(s), drug(s), biomarker application(s), Mascot peptide score, and peptide matches is presented for serum-derived EV (Table S2) and urine-derived EV (Table S3). All proteomic data are available for download at the ExoCarta database (www.exocarta.org) (Mathivanan et al. 2011; Simpson et al. 2012).

#### Proteomic analysis of serum-derived EV

**Fig. 2:**
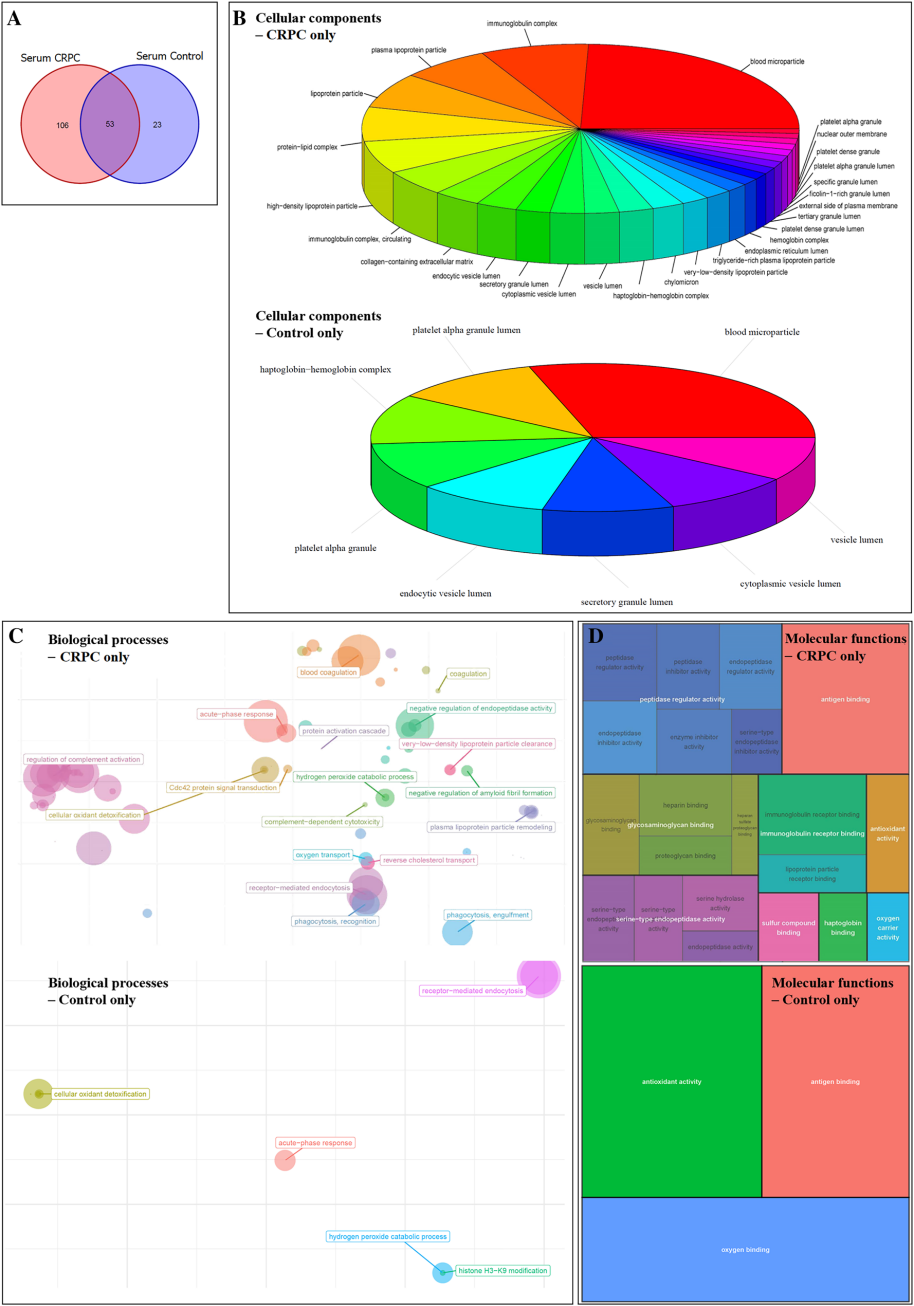
Proteomic analysis of CRPC and control serum-derived EV. **A** Venn diagram of the number of proteins identified in CRPC and control serum-derived EV. **B** Cellular component, **C** biological process, and **D** molecular function enrichment analyses of serum-derived EV proteins that are exclusive to the CRPC (*n* = 12) and control (*n* = 3) samples

Among the proteins identified in serum-derived EV, 23 and 106 proteins were exclusive to the control and CRPC groups, respectively, while 53 were common to both groups (Fig. 2A). Mutual proteins found in both groups (mainly normal serum proteins) were excluded from further analysis. Functional enrichment analysis was performed to identify GO terms statistically enriched by serum-derived proteins exclusive to the control and CRPC groups, and the enriched cellular components (Fig. 2B), biological processes (Fig. 2C), and molecular functions (Fig. 2D) were identified and visualised.

Cellular component analysis (Fig. 2B) showed that the major fraction of serum-derived EV proteins specific to both CRPC and control samples were primarily localised in blood microparticles, likely indicative of ubiquitous blood proteins. Aside from this primary fraction, other serum EV proteins derived from CRPC samples were mainly found in immunoglobulin complexes, lipoprotein particles, and protein-lipid complexes, while those specific to controls were mainly found in platelet alpha granules, haptoglobin–haemoglobin complexes, and vesicle lumens.

Biological process analysis (Fig. 2C) showed distinct differences between serum EV proteins from CRPC and control samples. Proteins from CRPC samples were mainly involved in a number of immune-related processes including blood coagulation, regulation of complement activation, and phagocytosis. Main biological processes common to both CRPC- and control-derived proteins included cellular oxidant detoxification, acute-phase response, and hydrogen peroxide catabolic process.

Molecular function analysis (Fig. 2D) showed that antigen binding was a common function of serum EV proteins from both CRPC and control samples. Other than this, CRPC-derived serum EV proteins exhibited main functions of heparin binding, peptidase regulator activity, and immunoglobulin receptor binding. In contrast, control-derived serum EV proteins exhibited main functions of antioxidant activity and oxygen binding, which were not strongly represented functions in the CRPC-derived proteins.

Overall, CC, BP, and MF analyses all showed a much more diverse range of components, processes or functions for the CRPC-derived serum EV proteins compared to the control-derived proteins.

#### Proteomic analysis of urine-derived EV

**Fig. 3:**
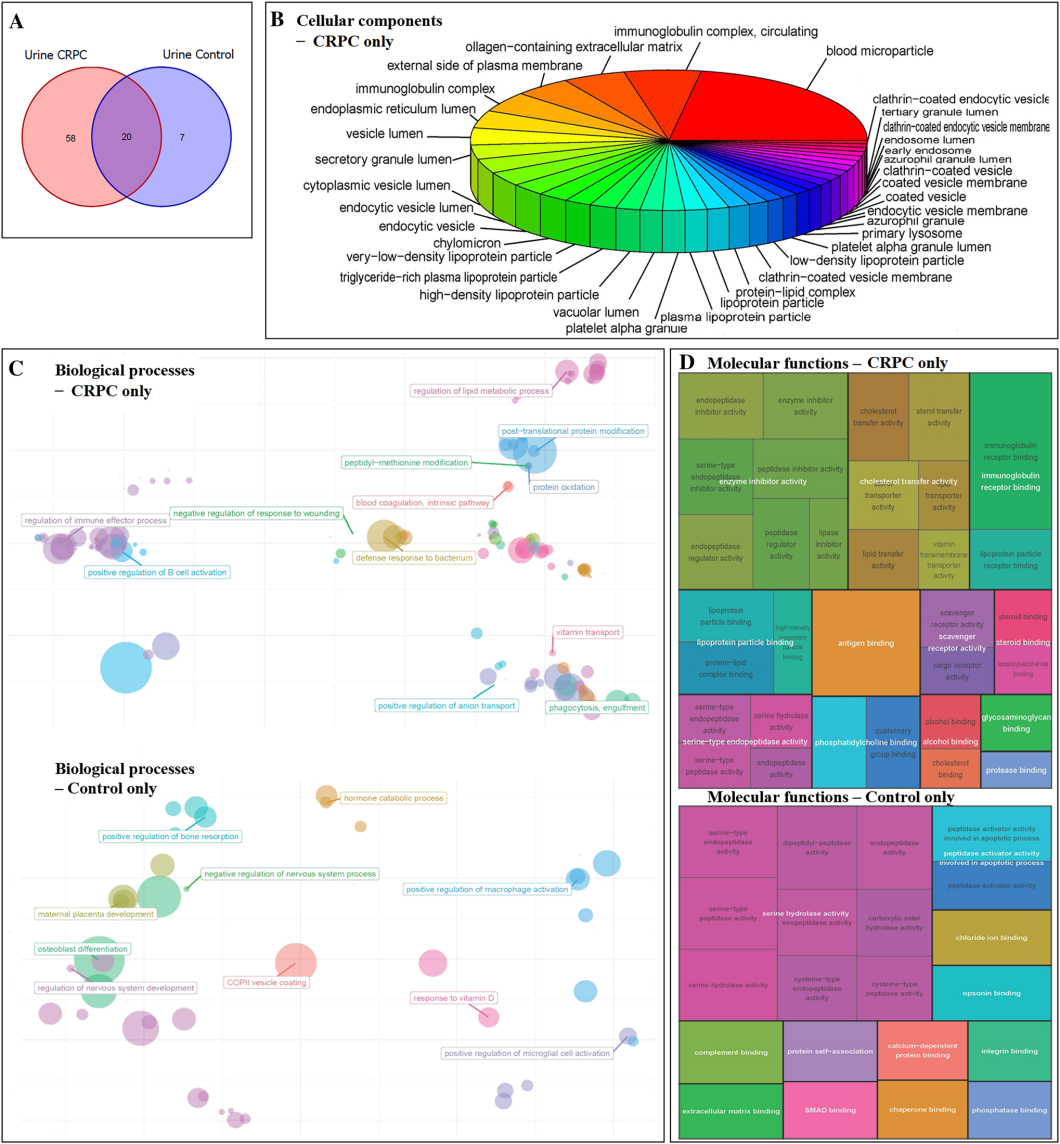
Proteomic analysis of CRPC and control urine-derived EV. **A** Venn diagram of the number of proteins identified in CRPC and control urine-derived EV. **B** Cellular component, **C** biological process, and **D** molecular function enrichment analyses of urine-derived EV proteins that are exclusive to the CRPC (*n* = 9) and control (*n* = 3) samples. Visualisation of cellular components is limited to CRPC urine-derived EV proteins since only one GO term was enriched for control urine-derived EV proteins (endoplasmic reticulum lumen, *p*-value = 0.0049, adjusted *p*-value = 0.088). GO terms enriched by 7 urine-derived EV proteins exclusive to controls were selected based on the significance level of adjusted *p*-value < 0.1

Among the proteins identified in urine-derived EV, 58 and 7 proteins were exclusive to the control and CRPC groups, respectively, while 20 were common to both groups (Fig. 3A). Mutual proteins found in both groups (mainly normal urine proteins) were excluded from further analysis. Functional enrichment analysis was performed to identify GO terms statistically enriched by urine-derived proteins exclusive to the control and CRPC groups, and the enriched cellular components (Fig. 3B), biological processes (Fig. 3C), and molecular functions (Fig. 3D) were identified and visualised.

There were 7 control-specific urine-derived EV proteins which could not statistically enrich any GO terms at the stringent cutoff of *p* < 0.05. Therefore, for the analysis of control-specific urine-derived EV proteins only, we relaxed the cutoff and investigated functions enriched at the significance level of adjusted *p*-value < 0.1.

Cellular component analysis (Fig. 3B) showed that the major fraction of urine-derived EV proteins specific to CRPC samples were primarily localised in blood microparticles, similar to what was observed for serum-derived EV proteins. Aside from this primary fraction, other urine EV proteins specific to CRPC were mainly found in immunoglobulin complexes, vesicle lumens, and lipoprotein particles.

Biological process analysis (Fig. 3C) showed distinct differences between urine EV proteins from CRPC and control samples. CRPC-derived proteins were mainly involved in a number of immune-related processes including blood coagulation, regulation of immune cells, defence response to pathogens, and phagocytosis. Control-derived proteins were mainly involved in a range of normal physiological processes such as bone- and nervous system-related processes.

Molecular function analysis (Fig. 3D) showed that the main functions of CRPC-derived urine EV proteins included enzyme inhibitor activity, cholesterol transfer activity, immunoglobulin receptor binding, and lipoprotein particle binding. Control-derived urine EV proteins exhibited functions mainly related to serine hydrolase activity.

#### Analysis of EV proteins common in serum and urine

**Fig. 4:**
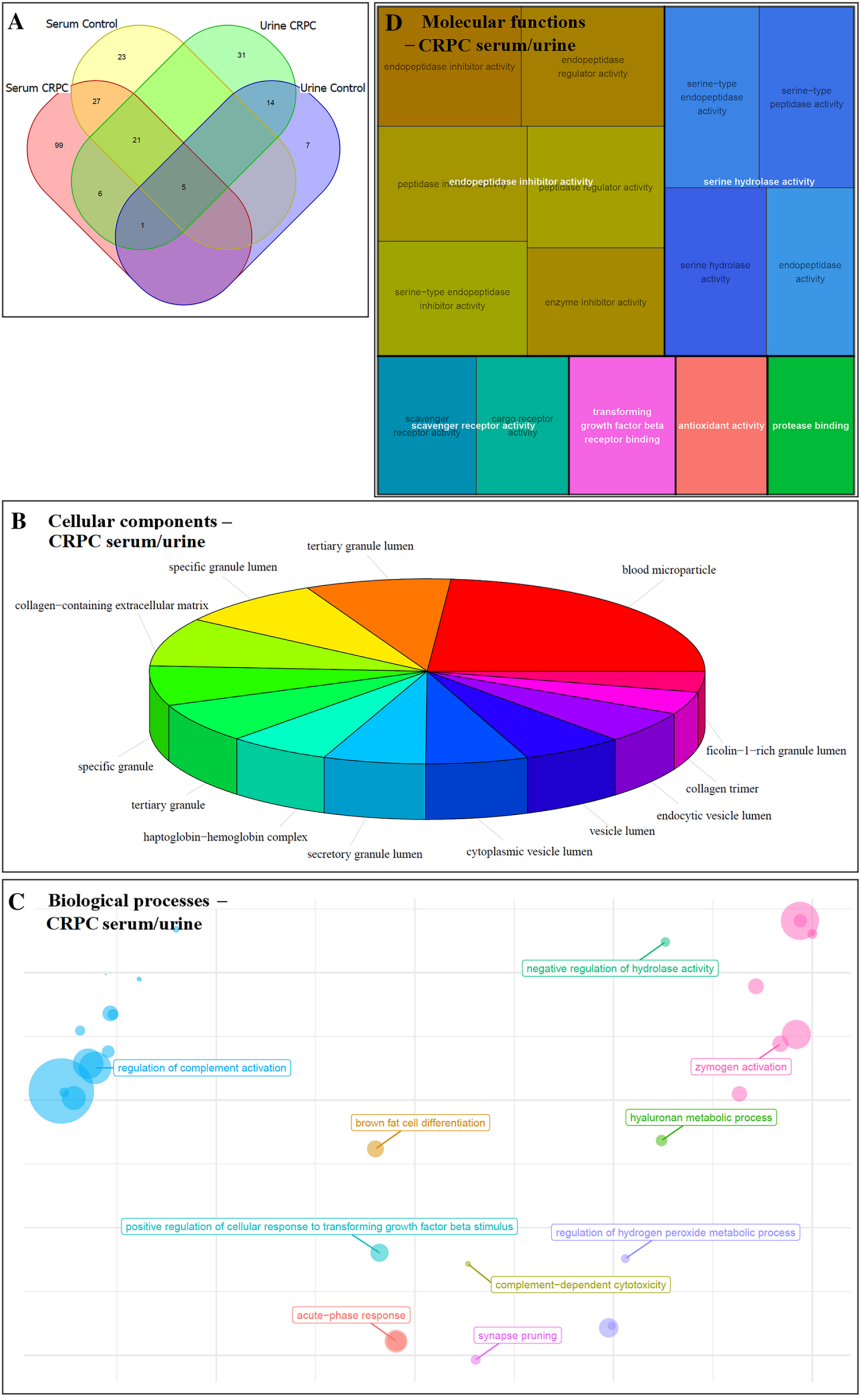
Analysis of EV proteins that were exclusively found in both serum and urine of CRPC samples. **A** Venn diagram showing the number of proteins identified in different sample groups. **B** Cellular component, **C** biological process, and **D** molecular function enrichment analyses of EV proteins that were exclusively found in both serum (*n* = 12) and urine (*n* = 9) of CRPC samples, and not present in control samples

Among all EV proteins identified from serum and urine, 5 were mutual between serum and urine controls, while 33 were mutual between serum and urine CRPC samples (Fig. 4A). Removing from these 33 proteins those that were also found in serum and urine controls, a total of 6 proteins were identified that were present in both serum and urine CRPC samples, but not in control samples. These proteins were: CD5 antigen-like protein, complement C1q C Chain, leucine-rich alpha-2-glycoprotein, pregnancy zone protein, haptoglobin, and inter-alpha-trypsin inhibitor heavy chain H2. Statistical enrichment analyses have revealed cellular components (Fig. 4B), biological processes (Fig. 4C), and molecular functions (Fig. 4D) overrepresented by these 6 EV proteins exclusive to both serum and urine CRPC samples (*adjusted p*-value < 0.05).

Despite some variations, the results of CC, BP, and MF analyses in Fig. 6 mostly overlapped with those shown in Figs. 2 and 3 for CRPC-derived proteins from serum and urine, respectively. An interesting point worth noting was that “transforming growth factor beta receptor binding” was a molecular function identified in Fig. 4, for EV proteins exclusively found in both serum and urine CRPC samples that was not seen in Figs. 2 and 3.

#### Matched patient samples

**Fig. 5:**
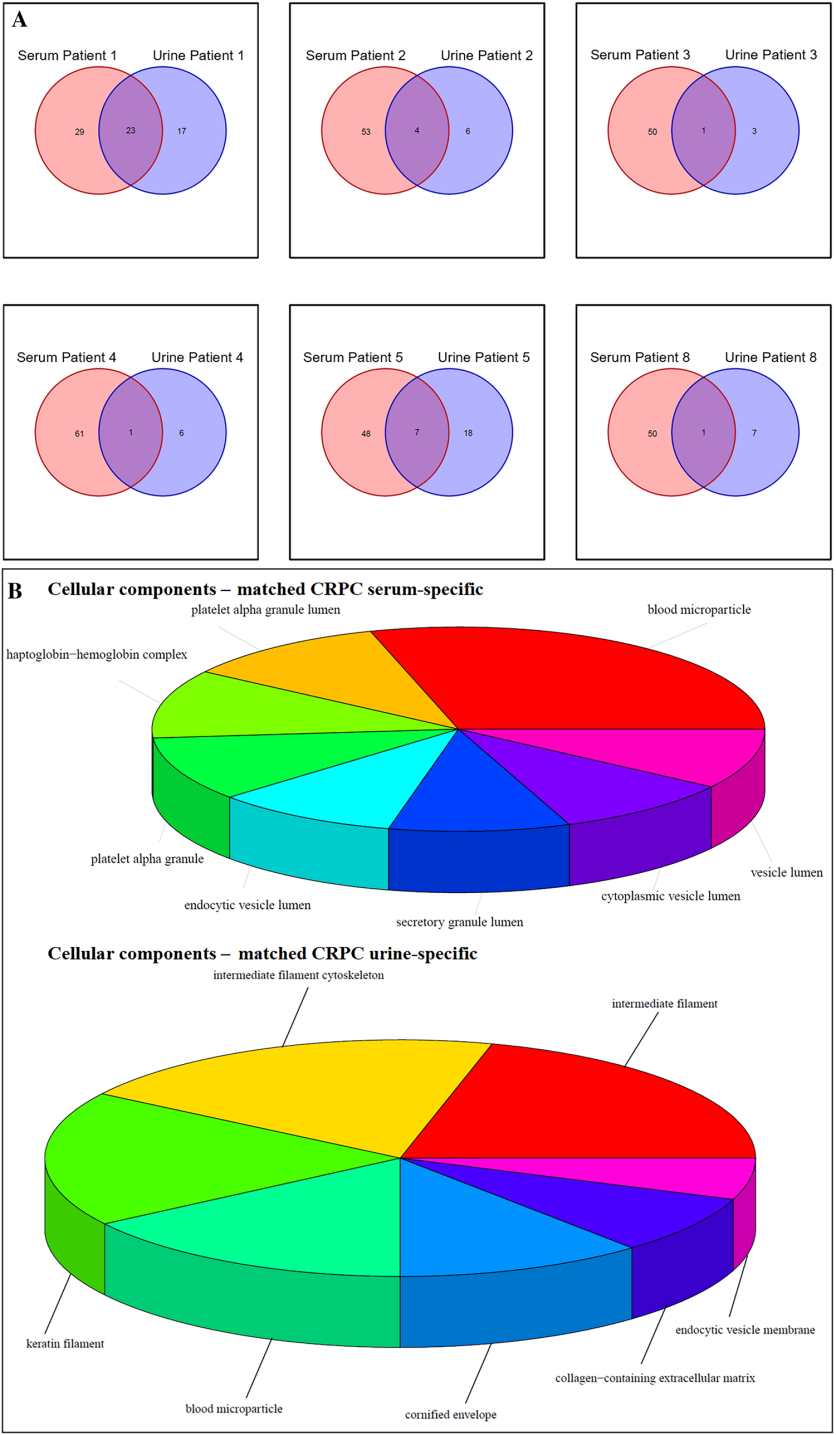

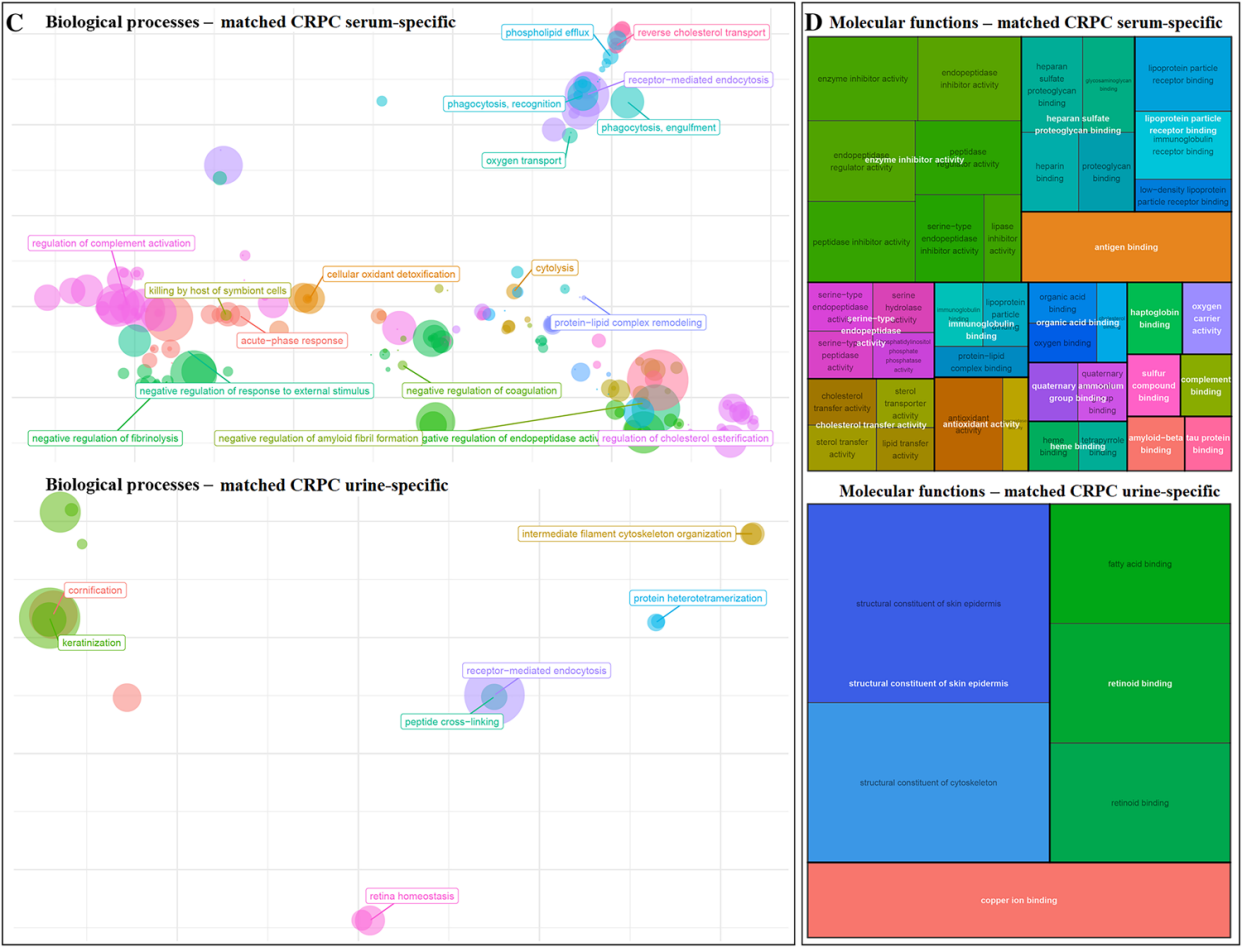
Analysis of EV proteins from matched serum and urine samples of 6 CRPC patients. **A** Venn diagram showing the number of proteins mutual and exclusive to serum and urine for each of the 6 patients with matched samples. **B** Cellular component, **C** biological process, and **D** molecular function enrichment analyses of EV proteins that were found uniquely in the serum and urine of the 6 patients with matched samples

Six CRPC patients provided matched serum and urine samples collected at the same time for our analysis. Except for one, most of these six patients had few EV proteins that were mutual between their individual serum and urine samples (Fig. 5A). Across these six patients, 74 EV proteins were specific to serum and 35 EV proteins were specific to urine. Statistical functional analysis on these two sets of genes were performed and significant terms (FDR < 0.05) including cellular components (Fig. 5B), biological processes (Fig. 5C), and molecular functions (Fig. 5D) were visualised. Maps showing the proteins found specifically in the serum (Figure S3) and urine (Figure S4) for each of these six patients are included as supplementary figures.

Despite some variations, the results of CC, BP, and MF analyses in Fig. 5 generally overlapped with those shown in Figs. 2, 3 and 4 for CRPC-derived proteins from serum and urine. It is worth noting that a much more diverse range of biological processes and molecular functions were identified for serum-specific EV proteins from the six matched samples compared to the urine-specific EV. Moreover, the identified processes and functions for serum-specific EV proteins were more likely related to CRPC pathology than the urine-specific EV proteins, which appeared to be related to generic physiological processes involved within the eye and skin.

### Analysis of cholesterol content

**Fig. 6:**
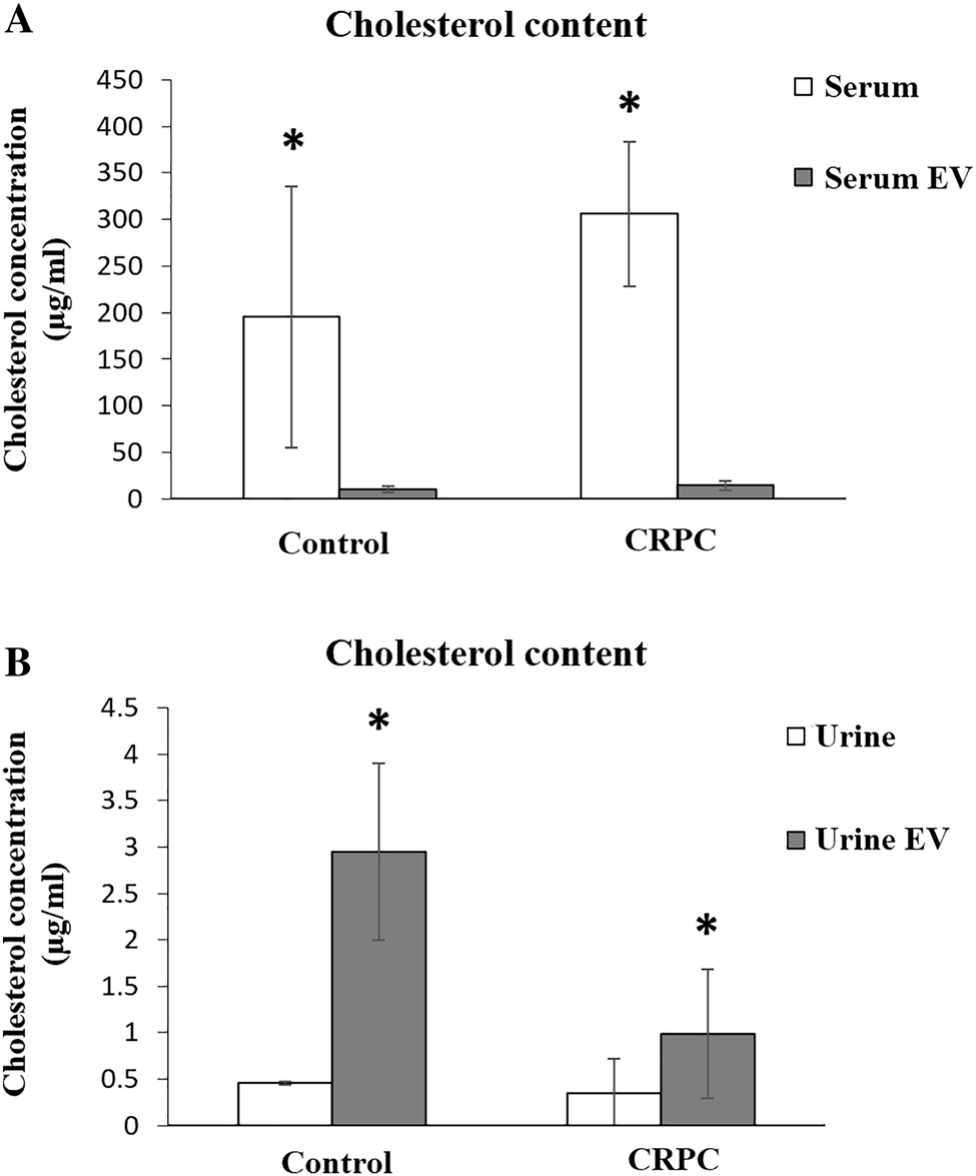
Cholesterol concentration of serum- and urine-derived EVs. Cholesterol concentration of **A** serum and serum-derived EVs (control *n* = 3, CRPC *n* = 12), and **B** urine and urine-derived EVs (control *n* = 3, CRPC *n* = 9). *p < 0.05

The cholesterol content of all samples (serum, urine, and their isolated EV) was determined using LC–MS. Compared to whole serum, the serum-derived EV had significantly lower cholesterol concentration in both control and CRPC groups (Fig. 6A). The average cholesterol level of serum-derived EV was slightly higher in the CRPC group (14.50 μg/mL) compared to the control (10.75 μg/mL). Interestingly, the urine-derived EV were enriched in cholesterol compared to whole urine in both groups (Fig. 6B). The average cholesterol level of urine-derived EV was significantly lower in the CRPC group (0.96 μg/mL) compared to the control (2.95 μg/mL).

## Discussion

EV derived from cancer cells contain proteins, lipids, and genetic markers that can provide indicators for cancer development and progression. Due to their presence in easily accessible biological fluids, EV are being exploited as a potential source of cancer biomarkers or therapeutic targets (Wu et al. 2019). In this study, we used mass spectrometry-based proteomic analysis to characterise EV from serum and urine samples of CRPC patients compared to healthy controls. In the context of the existing literature on liquid biopsy-based identification of PCa markers from EV (Pang et al. 2020; Ramirez-Garrastacho et al. 2021), our study was unique in several aspects as follows: 1) we compared the proteomic content of CRPC serum- and urine-derived EV, including from six patients who provided matched serum and urine samples collected at the same time, 2) we were able to increase the yield of EV from the biological fluid samples by removing albumin (> 90%) during sample processing, and 3) we demonstrated interesting trends in the cholesterol content of urine-derived EV.

In this study, EV were isolated from serum and urine using the sucrose assisted differential centrifugation method. TEM, NTA, and western blot analyses confirmed the successful isolation of homogeneous EV from both types of biological fluids, through EV morphology and size resembling classic exosomes, as well as the presence and enrichment of exosomal markers. Exosome isolation from biological fluids has been a persisting challenge in EV research. While differential centrifugation can reduce the presence of contamination, it cannot eliminate the co-sedimentation of other vesicles, platelet-derived microparticles (in plasma) (Siljander 2011), non-EV lipid particles, or protein aggregates with a similar size range as the EV targeted for isolation. Additional steps, such as filtration or the inclusion of sucrose gradients, have improved the purity of EV isolation to some degree, although often at the cost of yield (Choi et al. 2015). Further complexity is introduced by the limited volume of clinical samples, as well as the abundance of ubiquitous proteins such as albumin in serum and uromodulin in urine. In this study, we used AlbuminOUT™ and DTT during sample preparation, which significantly reduced the levels of highly abundant proteins in serum and urine (> 90% clearance). However, due to the inability to achieve 100% clearance, the possible co-presence of other EV and secretory proteins in the isolated EV should be considered when interpreting the results of our analyses. Until more efficient and reproducible EV isolation protocols are developed for biological fluid samples, the limitations of current methodologies need to be recognised.

While serum-derived EV can potentially originate from any tissue in the body, urine-derived EV are mainly derived from the kidney, bladder, seminal vesicle, prostate, urethra, or immune cell infiltrate (Drake and Kislinger 2014). Our results demonstrated that the proteomic profiles of serum- and urine-derived EV in CRPC patients differed from those of healthy controls and also from each other. Such differences extend beyond proteomic profiles, as reported in other studies (Joncas et al. 2019; Matsuzaki et al. 2021; Davey et al. 2020), pointing to the potential of identifying PCa-specific markers from biological fluid-derived EV. We have insufficient evidence to understand whether these differences are treatment dependent, prompting the need for further studies to provide meaningful insights into utilising differentially expressed markers as indicators of disease progression or treatment response. Interestingly, the serum-derived EV from CRPC patients showed a statistically significant difference in both number and size compared to those from controls, while no such differences were observed for urine-derived EV. Similarly, other studies reported that the level of plasma-derived EV was significantly higher in PCa patients when compared to healthy donors (Lázaro-Ibáñez et al. 2014; Tavoosidana et al. 2011). In ovarian cancer patients, the level of circulating EV was reported to increase with cancer stage (Taylor and Gercel-Taylor 2008), suggesting that cancer cells not only secrete higher levels of EV into the blood, but their level of EV secretion also increases with disease progression. One possible mechanism is that in response to stress, tumour suppressor protein p53 induces the function of endosomes, which regulate the transcription of tumour-suppressor activated pathway-6 (TSAP-6) to enhance EV production (Yu et al. 2006). It is also possible that the mutation and upregulation of the Rab GTPase family in cancer cells characteristically promotes EV secretion (Ostrowski et al. 2010; Gopal Krishnan et al. 2020).

Previous studies by our team (Hosseini-Beheshti et al. 2012) and others (Duijvesz et al. 2013) reporting proteomic analyses of EV isolated from a range of PCa cell lines have identified over 200 proteins, of which approximately 50 were identified as protein biomarkers that were not present in EV isolated from benign/control cell lines. Interestingly, there was not a high degree of overlap in the proteins identified from the biological fluids of CRPC patients in this study compared to those previously identified from PCa cell lines. For instance, only 6 proteins were mutually observed in this and our previous study (Hosseini-Beheshti et al. 2012). Possible explanations include differences in the source of EV, as well as isolation protocol used for clinical samples compared to PCa cell culture-derived samples.

In this study, we identified 182 proteins overall in serum-derived EV and 85 proteins overall in urine-derived EV, among all CRPC and control samples, and observed some overlap between serum- and urine-derived EV proteins. Over 20 of these proteins have been reported in other proteomic studies on EV derived from non-PCa blood and urine samples, including hemopexin, lysosomal-associated membrane protein 2, regulator of G-protein signaling 20, complement component 3, alpha-2-macroglobulin, CD14, CD59, ceruloplasmin, vitamin D binding protein, haptoglobin, heterogeneous nuclear ribonucleoprotein M, inter-alpha-trypsin inhibitor heavy chain 2, leucine-rich alpha-2-glycoprotein 1, mannan-binding lectin serine peptidase 2, phosphoinositide-3-kinase interacting protein 1, and transferrin (Bernardino et al. 2021; Karimi et al. 2018; Merchant et al. 2017). In terms of putative markers for the detection of PCa, our results had some agreement with another study that derived candidate urine biomarkers for PCa by mining cancer genomic profiles from public databases (Chen et al. 2011). Compared to this study, the mutually identified proteins in our proteomic data were immunoglobulin superfamily member 8 (in both blood and urine), apolipoprotein D (urine), complement factor H (blood), and retinol binding protein 4 (urine). Interestingly, our data had little overlap compared to a few recent investigations on the proteomic profile of EVs derived from urine samples of PCa patients using LC–MS. These studies identified transmembrane protein 256 and LAMTOR1 (Øverbye et al. 2015), flotillin 2 and Rab3B (Wang et al. 2017), FABP5 (Fujita et al. 2017), among others (Fujita et al. 2017; Sequeiros et al. 2017) as PCa-specific urine biomarkers. The inconsistency in urine marker identification among these studies and when compared to our data perhaps reflects the need for further studies and varying approaches to EV isolation in these samples (Wang et al. 2022). As a source for protein-based biomarker discovery, urine remains a challenging biological fluid to handle. Large volumes may be required to capture a detectable level of protein candidates secreted from the prostate as the urine passes through, while an overall low protein concentration and the presence of certain salts in the urine are further impediments (Bernardino et al. 2021; Sequeiros et al. 2017). It is possible that PCa-specific EV are not as well concentrated in urine compared to serum, suggesting that urine may not be an optimal type of biological fluid sample for the detection of PCa markers.

Our study was unique in analysing EV proteins derived from both serum and urine of CRPC patients. Overall, EV proteins from CRPC serum samples showed diverse results of CC, BP, and MP analyses and many of the components, processes, and functions identified could be related to the dysregulation of immunological processes in PCa (Vitkin et al. 2019). In contrast, our findings for EV proteins from CRPC urine samples identified a small number of normal physiological processes and functions. These observations were verified by our analysis of serum- and urine-specific EV proteins from matched samples of six CRPC patients—another unique point of our study. Interestingly, from our overall analysis, we identified 6 EV proteins that were exclusively found in *both* serum and urine of CRPC patients and were not present in control serum or urine samples. These proteins were CD5 antigen-like protein, complement C1q C Chain, leucine-rich alpha-2-glycoprotein, pregnancy zone protein, haptoglobin, and inter-alpha-trypsin inhibitor heavy chain H2. It is evident that these six EV proteins are associated with important immunoregulatory and apoptotic mechanisms, which could be affected in PCa. Moreover, their MF analysis revealed transforming growth factor (TGF)-β receptor binding as a major function, which was not seen in separate MF analyses of CRPC serum-derived or urine-derived EV proteins. This finding points to the TGF-β mechanistic pathway in PCa progression (Li et al. 2008) and warrants further investigation as a therapeutic target by controlling upstream molecular regulators (Huang et al. 2019).

Our group has previously shown evidence of de novo androgen synthesis from cholesterol precursors in the local tumour microenvironment, suggesting that cholesterol-associated mechanisms can contribute to CRPC (Locke et al. 2008), which may be supported by the presence of CYP17 in exosomes derived from human serum (Locke et al. 2009). In line with these observations, we reported that EV derived from PCa cell lines contained significantly higher cholesterol levels than the control benign cell line (Hosseini-Beheshti et al. 2012). While the cholesterol content of serum-derived EV in our study did not yield significant findings, we did observe that the cholesterol levels in CRPC urine-derived EV were significantly lower than in the control group. Furthermore, there was significant enrichment of cholesterol in the urine-derived EV of both CRPC and control groups compared to in whole urine samples. These findings are consistent with our previous conclusions regarding a possible role of cholesterol in CRPC (Hosseini-Beheshti et al. 2012), as well as with others observing significant differences in the levels of several lipid species in EV derived from urine of prostate cancer patients compared to healthy controls (Skotland et al. 2017). While further studies are needed to draw definitive conclusions, a correlative decrease in the cholesterol content of urinary EV may be of interest to pursue as a potential indicator of CRPC.

There are some limitations relating to our selection of control patient samples that should be considered when interpreting the results of our study. We opted to select younger donors for our control samples, rather than to age-match with a control cohort of similar age (57–81 years old) as the CRPC patients. This was to avoid the significant risk of having control patients who may be undiagnosed with asymptomatic prostatic disease or cancer in older age groups, which would invalidate our findings. Notably, the prevalence of undiagnosed PCa at autopsy is estimated at 36% for Caucasians and 51% for African-Americans among men aged 70–79 (Jahn et al. 2015). We have, therefore, used a younger control cohort who are well outside the age range of having significant risk of a prostatic condition, such that we can have greater confidence on the CRPC markers identified in this study. Although potential differences exist in global protein expression between the control and CRPC samples due to age, these difference are likely related to normal physiological processes (Jahn et al. 2015) rather than affecting PCa-related marker expression. In addition, the control group in our study had a relatively small sample size. This was a compromise to the novelty of our study, to have matched serum and urine samples from the control group, which has not been reported in other studies on EV proteomic analysis in the context of PCa. It is worth noting that other studies on EV analysis in PCa have used a similar sample size for their control cohort (*n* < 5) (Lázaro-Ibáñez et al. 2014; Fujita et al. 2017). A smaller sample size increases the potential impact of biological variation, but these variations are expected to have greater impact on normal physiological processes rather than the identification of PCa markers. In this study, we have specifically compared the EV proteomic cargo of CRPC patients treated by Abiraterone with healthy controls, using biological fluid samples derived from a larger clinical trial. It is imperative to state that to conduct a robust search for PCa-specific biomarkers, we need to include patient control samples from varying stages of cancer progression. In addition to a healthy cohort, we need to include untreated primary PCa, as well as treated androgen dependent and CRPC patients.

## Conclusion

The characterisation of biological fluid-derived EV for the purpose of PCa biomarker discovery holds an encouraging future. In this study, we established a platform for analysing the proteomic profile and cholesterol content of EV derived from serum and urine samples of CRPC patients, including by using matching biological fluid samples from the same patients. We found significant differences when comparing data between CRPC patients and healthy controls, indicating the potential to adopt EV proteins derived from biological fluids as PCa markers. Future studies will be necessary, along with improvements in EV isolation techniques, to develop a highly multiplexed and targeted proteomic and lipidomic assessment of EV-enriched clinical specimens for PCa biomarker discovery.

## Supplementary Information

Below is the link to the electronic supplementary material.

Supplementary file1 (DOCX 4193 KB)

## Data Availability

The datasets generated and/or analysed during the current study are available from the corresponding author on reasonable request.
