# Supplementary material for: Extracellular vesicles from biological fluids as potential markers in castration resistant prostate cancer
Source: J Cancer Res Clin Oncol. 2022 Oct 12;149(8):4701–17. doi: 10.1007/s00432-022-04391-6 (PMC10349738; doi:10.1007/s00432-022-04391-6)

**Table S1.** Patient information. PSA levels were measured at baseline at the time of study entry and before starting Abiraterone. The patients recruited to this research study received Abiraterone in the second or higher treatment setting (counting Casodex as a first line therapy). The patient’s treatment history informs the biological status of the patient at the time they were enrolled into the clinical trial from which patient samples were derived for this study.

| **Patient** | **Age** | **PSA (ng/ml)** | **Treatment History** |
| --- | --- | --- | --- |
| 1 | 79 | 39.12 | Casodex |
| 2 | 65 | 60.21 | Flutamide, Docetaxel |
| 3 | 73 | 17.5 | Casodex, Corticosteroid, Flutamide |
| 4 | 60 | 76.85 | Casodex, Corticosteroid |
| 5 | 69 | 151.36 | Casodex |
| 6 | 70 | 253.14 | Casodex, Docetaxel |
| 7 | 74 | 39.65 | Casodex, Corticosteroid, ketoconazole, Carbotaxol, Taxotere |
| 8 | 80 | 227.36 | Casodex, Docetaxel |
| 9 | 57 | 8.56 | Casodex, Corticosteroid, Flutamide |
| 10 | 73 | 12.16 | Casodex, Docetaxel |
| 11 | 81 | 32.16 | Decetaxel |
| 12 | 73 | 20.23 | Casodex, Docetaxel |
| 13 | 65 | 281.76 | Casodex |
| 14 | 72 | 29.4 | Casodex |
| 15 | 78 | 33.9 | Casodex, Corticosteroid, ketoconazole, Docetaxel |

**Table S2.** Proteins identified in serum-derived EV from PCa patients and controls.

| **ID** | **Protein Name** | **Subcellular location** | **Type(s)** | **Drug(s)** | **Serum exosomes** | | **Biomarker Application(s)** | **Mascot score** | **Peptide matches** |
| --- | --- | --- | --- | --- | --- | --- | --- | --- | --- |
|  |  |  |  |  | **Control** | **PCa** |  |  |  |
| **P04217** | alpha-1-B glycoprotein | Extracellular Space | other |  | **+** | **+** |  | 173 | 18 |
| **P01023** | alpha-2-macroglobulin | Extracellular Space | transporter |  | **+** | **+** |  | 227 | 21 |
| **P01019** | angiotensinogen (serpin peptidase inhibitor, clade A, member 8) | Extracellular Space | growth factor |  | **+** | **+** | efficacy | 89 | 11 |
| **P02768** | albumin | Extracellular Space | transporter |  | **+** | **+** | diagnosis, efficacy, prognosis, safety, unspecified application | 168 | 21 |
| **P02647** | apolipoprotein A-I | Extracellular Space | transporter |  | **+** | **+** | diagnosis, efficacy, unspecified application | 129 | 17 |
| **P02652** | apolipoprotein A-II | Extracellular Space | transporter |  | **+** | **+** |  | 165 | 23 |
| **P04114** | apolipoprotein B | Extracellular Space | transporter | mipomersen | **+** | **+** | diagnosis, efficacy | 137 | 15 |
| **P01024** | complement component 3 | Extracellular Space | peptidase | IgG | **+** | **+** | efficacy | 136 | 16 |
| **P0C0L4** | complement component 4B (Chido blood group) | Extracellular Space | other | IgG | **+** | **+** |  | 171 | 19 |
| **P04003** | complement component 4 binding protein, alpha | Extracellular Space | other |  | **+** | **+** |  | 105 | 12 |
| **O75155-2** | cullin-associated and neddylation-dissociated 2 (putative) | Nucleus | transcription regulator |  | **+** |  |  | 14 | 1 |
| **P00450** | ceruloplasmin (ferroxidase) | Extracellular Space | enzyme |  | **+** | **+** | efficacy | 84 | 9 |
| **A8K855-2** | EF-hand calcium binding domain 7 | Other | other |  | **+** |  |  | 35 | 1 |
| **Q9NY74** | Ewing tumor-associated antigen 1 | Other | other |  | **+** |  |  | 16 | 2 |
| **P02671-2** | fibrinogen alpha chain | Extracellular Space | other | F2 | **+** |  | diagnosis | 124 | 7 |
| **P68871** | hemoglobin, beta | Cytoplasm | transporter | iron dextran | **+** | **+** |  | 114 | 12 |
| **P02042** | hemoglobin, delta | Other | transporter |  | **+** | **+** |  | 32 | 4 |
| **P00738** | haptoglobin | Extracellular Space | peptidase |  | **+** | **+** | diagnosis, efficacy, unspecified application | 166 | 20 |
| **P00739** | haptoglobin-related protein | Extracellular Space | peptidase |  | **+** | **+** |  | 106 | 12 |
| **P02790** | hemopexin | Extracellular Space | transporter |  | **+** | **+** |  | 151 | 17 |
| **P04196** | histidine-rich glycoprotein | Extracellular Space | other |  | **+** | **+** |  | 105 | 11 |
| **P01876** | immunoglobulin heavy constant alpha 1 | Extracellular Space | other |  | **+** | **+** |  | 136 | 17 |
| **P01877** | immunoglobulin heavy constant alpha 2 (A2m marker) | Extracellular Space | other |  | **+** | **+** |  | 129 | 29 |
| **P01857** | immunoglobulin heavy constant gamma 1 (G1m marker) | Extracellular Space | other |  | **+** | **+** |  | 155 | 17 |
| **P01859** | immunoglobulin heavy constant gamma 2 (G2m marker) | Plasma Membrane | other |  | **+** | **+** |  | 122 | 15 |
| **P01860** | immunoglobulin heavy constant gamma 3 (G3m marker) | Extracellular Space | other |  | **+** | **+** |  | 146 | 16 |
| **P01861** | immunoglobulin heavy constant gamma 4 (G4m marker) | Extracellular Space | other |  | **+** | **+** |  | 121 | 14 |
| **P01871-2** | immunoglobulin heavy constant mu | Plasma Membrane | transmembrane receptor |  | **+** | **+** |  | 164 | 18 |
| **P01834** | immunoglobulin kappa constant | Extracellular Space | other |  | **+** | **+** | unspecified application | 144 | 14 |
| **P0CG05** | immunoglobulin lambda constant 2 (Kern-Oz- marker) | Extracellular Space | other |  | **+** | **+** |  | 121 | 14 |
| **P02763** | orosomucoid 1 | Extracellular Space | other |  | **+** | **+** | unspecified application | 132 | 12 |
| **P19652** | orosomucoid 2 | Extracellular Space | other |  | **+** | **+** |  | 122 | 13 |
| **P00747** | plasminogen | Extracellular Space | peptidase | tenecteplase, PLAT, tranexamic acid, aprotinin, 6-aminocaproic acid, reteplase | **+** |  |  | 17 | 1 |
| **P01009-2** | serpin peptidase inhibitor, clade A (alpha-1 antiproteinase, antitrypsin), member 1 | Extracellular Space | other |  | **+** | **+** | diagnosis, unspecified application | 108 | 13 |
| **P01008** | serpin peptidase inhibitor, clade C (antithrombin), member 1 | Extracellular Space | enzyme | heparin, enoxaparin, ardeparin, SR-123781A, glucuronyl glucosamine glycan sulfate, fondaparinux, nadroparin | **+** |  |  | 18 | 1 |
| **Q149N8-4** | SNF2 histone linker PHD RING helicase, E3 ubiquitin protein ligase | Nucleus | transcription regulator |  | **+** |  |  | 23 | 1 |
| **Q5JUK2-2** | spermatogenesis and oogenesis specific basic helix-loop-helix 1 | Cytoplasm | transcription regulator |  | **+** |  |  | 14 | 2 |
| **P02549-2** | spectrin, alpha, erythrocytic 1 (elliptocytosis 2) | Cytoplasm | other |  | **+** |  |  | 29 | 1 |
| **Q8NHU6-2** | tudor domain containing 7 | Cytoplasm | other |  | **+** | **+** |  | 21 | 1 |
| **P02787** | transferrin | Extracellular Space | transporter |  | **+** | **+** | efficacy, prognosis | 110 | 12 |
| **Q15361** | transcription termination factor, RNA polymerase I | Nucleus | transcription regulator |  | **+** |  |  | 19 | 1 |
| **P04004** | vitronectin | Extracellular Space | other |  | **+** | **+** |  | 112 | 12 |
| **Q96GC6-4** | zinc finger protein 274 | Nucleus | transcription regulator |  | **+** |  |  | 26 | 2 |
| **Q68DY1-2** | zinc finger protein 626 | Other | other |  | **+** |  |  | 18 | 8 |
| **P02747** | complement component 1, q subcomponent, C chain | Extracellular Space | other |  |  | **+** |  | 75 | 10 |
| **P01031** | complement component 5 | Extracellular Space | cytokine | eculizumab, IgG |  | **+** |  | 94 | 11 |
| **O75808** | calpain 15 | Other | peptidase |  |  | **+** |  | 15 | 1 |
| **O43866** | CD5 molecule-like | Plasma Membrane | transmembrane receptor |  |  | **+** |  | 165 | 16 |
| **P08603** | complement factor H | Extracellular Space | other |  |  | **+** | unspecified application | 45 | 14 |
| **P50461** | cysteine and glycine-rich protein 3 (cardiac LIM protein) | Nucleus | other |  |  | **+** |  | 17 | 1 |
| **A7MBM2** | dispatched homolog 2 (Drosophila) | Other | other |  |  | **+** |  | 14 | 1 |
| **O14490-5** | discs, large (Drosophila) homolog-associated protein 1 | Plasma Membrane | other |  |  | **+** |  | 24 | 2 |
| **Q96MC2** | dynein regulatory complex subunit 1 homolog (Chlamydomonas) | Extracellular Space | other |  |  | **+** |  | 14 | 1 |
| **A4FU69-2** | EF-hand calcium binding domain 5 | Other | other |  |  | **+** |  | 14 | 2 |
| **P00734** | coagulation factor II (thrombin) | Extracellular Space | peptidase | enoxaparin, desirudin, dabigatran etexilate, Fibrinogen, ximelagatran, antithrombin alfa, aspirin/dabigatran etexilate, dabigatran, argatroban, bivalirudin, lepirudin |  | **+** | diagnosis, unspecified application | 93 | 11 |
| **O95837** | guanine nucleotide binding protein (G protein), alpha 14 | Plasma Membrane | enzyme |  |  | **+** |  | 14 | 2 |
| **Q02846** | guanylate cyclase 2D, membrane (retina-specific) | Plasma Membrane | kinase |  |  | **+** |  | 14 | 1 |
| **P0CG04** | immunoglobulin lambda constant 1 (Mcg marker) | Cytoplasm | other |  |  | **+** |  | 298 | 4 |
| **Q8IWB1** | inositol 1,4,5-trisphosphate receptor interacting protein | Extracellular Space | other |  |  | **+** |  | 19 | 1 |
| **O43272-1** | proline dehydrogenase (oxidase) 1 | Cytoplasm | enzyme |  |  | **+** | diagnosis | 14 | 1 |
| **P08519** | lipoprotein, Lp(a) | Extracellular Space | other |  |  | **+** | efficacy | 45 | 14 |
| **P47972** | neuronal pentraxin II | Extracellular Space | other |  |  | **+** | diagnosis | 14 | 2 |
| **Q8NG80** | olfactory receptor, family 2, subfamily L, member 5 | Plasma Membrane | other |  |  | **+** |  | 31 | 1 |
| **P20742** | pregnancy-zone protein | Extracellular Space | other |  |  | **+** |  | 125 | 4 |
| **Q9NWS8-2** | required for meiotic nuclear division 1 homolog (S. cerevisiae) | Cytoplasm | other |  |  | **+** |  | 25 | 1 |
| **P0DJI8** | serum amyloid A1 | Extracellular Space | transporter |  |  | **+** | diagnosis, unspecified application | 85 | 13 |
| **O14907** | Tax1 (human T-cell leukemia virus type I) binding protein 3 | Cytoplasm | transcription regulator |  |  | **+** | unspecified application | 23 | 1 |
| **Q96PF1** | transglutaminase 7 | Other | enzyme |  |  | **+** |  | 31 | 2 |
| **O75157-2** | TSC22 domain family, member 2 | Extracellular Space | other |  |  | **+** |  | 15 | 1 |
| **Q99856** | AT rich interactive domain 3A (BRIGHT-like) | Nucleus | transcription regulator |  |  | **+** |  | 19 | 1 |
| **P02750** | leucine-rich alpha-2-glycoprotein 1 | Extracellular Space | other |  |  | **+** |  | 87 | 13 |
| **Q0VAA2** | leucine rich repeat containing 74 | Other | other |  |  | **+** |  | 17 | 1 |
| **P46199** | mitochondrial translational initiation factor 2 | Cytoplasm | translation regulator |  |  | **+** |  | 21 | 1 |
| **Q6IFG1** | olfactory receptor, family 52, subfamily E, member 8 | Plasma Membrane | G-protein coupled receptor |  |  | **+** |  | 21 | 2 |
| **P06702** | S100 calcium binding protein A9 | Cytoplasm | other |  |  | **+** | diagnosis, unspecified application | 61 | 10 |
| **P0DJI9** | serum amyloid A2 | Extracellular Space | other |  |  | **+** |  | 84 | 11 |
| **O75533** | splicing factor 3b, subunit 1, 155kDa | Nucleus | other |  |  | **+** |  | 27 | 2 |

**Table S3.** Proteins identified in urine-derived EV from PCa patients and controls.

| **ID** | **Protein Name** | **Subcellular location** | **Type(s)** | **Drug(s)** | **Urine exosomes** | | **Biomarker Application(s)** | **Mascot score** | **Peptide matches** |
| --- | --- | --- | --- | --- | --- | --- | --- | --- | --- |
|  |  |  |  |  | **Control** | **PCa** |  |  |  |
| **P02768** | albumin | Extracellular Space | transporter |  | **+** | **+** | diagnosis, efficacy, prognosis, safety, unspecified application | 150 | 18 |
| **P02760** | alpha-1-microglobulin/  bikunin precursor | Extracellular Space | transporter |  | **+** | **+** | safety, unspecified application | 97 | 17 |
| **H0Y4T9** | ankyrin repeat domain 26 | Nucleus | transcription regulator |  | **+** |  |  |  |  |
| **C9JF17** | apolipoprotein D | Extracellular Space | transporter |  | **+** | **+** | safety | 56 | 7 |
| **C9JEV0** | alpha-2-glycoprotein 1, zinc-binding | Extracellular Space | transporter |  | **+** |  |  | 81 | 14 |
| **P53634** | cathepsin C | Cytoplasm | peptidase |  | **+** |  | unspecified application | 13 | 1 |
| **P01859** | immunoglobulin heavy constant gamma 2 (G2m marker) | Plasma Membrane | other |  | **+** | **+** |  | 21 | 1 |
| **P01834** | immunoglobulin kappa constant | Extracellular Space | other |  | **+** | **+** | unspecified application | 132 | 13 |
| **P04264** | keratin 1 | Cytoplasm | other |  | **+** | **+** | diagnosis | 81 | 9 |
| **P13645** | keratin 10 | Cytoplasm | other |  | **+** |  |  | 80 | 8 |
| **P35908** | keratin 2 | Other | other |  | **+** |  |  | 67 | 9 |
| **P02538** | keratin 6A | Other | other |  | **+** |  | diagnosis | 77 | 9 |
| **P04259** | keratin 6B | Cytoplasm | other |  | **+** |  | diagnosis | 66 | 9 |
| **Q86Y46** | keratin 73 | Extracellular Space | other |  | **+** |  |  |  |  |
| **P35527** | keratin 9 | Other | other |  | **+** |  | diagnosis | 47 | 5 |
| **O00187-2** | mannan-binding lectin serine peptidase 2 | Extracellular Space | peptidase |  | **+** |  | prognosis | 22 | 1 |
| **P02763** | orosomucoid 1 | Extracellular Space | other |  | **+** | **+** | unspecified application | 98 | 10 |
| **P19652** | orosomucoid 2 | Extracellular Space | other |  | **+** | **+** |  | 66 | 9 |
| **H0Y5A1** | prostaglandin D2 synthase 21kDa (brain) | Cytoplasm | enzyme |  | **+** | **+** | efficacy | 59 | 8 |
| **D6RCM9** | RAN binding protein 3-like | Other | other |  | **+** |  |  | 19 | 1 |
| **P07998** | ribonuclease, RNase A family, 1 (pancreatic) | Extracellular Space | enzyme |  | **+** | **+** |  | 43 | 7 |
| **Q9HAT2** | sialic acid acetylesterase | Cytoplasm | enzyme |  | **+** |  |  | 28 | 1 |
| **P10451-2** | secreted phosphoprotein 1 | Extracellular Space | cytokine |  | **+** |  | diagnosis, efficacy, unspecified application | 38 | 2 |
| **P01023** | alpha-2-macroglobulin | Extracellular Space | transporter |  |  | **+** |  | 82 | 9 |
| **P02647** | apolipoprotein A-I | Extracellular Space | transporter |  |  | **+** | diagnosis, efficacy, unspecified application | 102 | 11 |
| **P02652** | apolipoprotein A-II | Extracellular Space | transporter |  |  | **+** |  | 37 | 8 |
| **P04114** | apolipoprotein B | Extracellular Space | transporter | mipomersen |  | **+** | diagnosis, efficacy | 109 | 15 |
| **P25311** | alpha-2-glycoprotein 1, zinc-binding | Extracellular Space | transporter |  |  | **+** |  | 79 | 10 |
| **P02747** | complement component 1, q subcomponent, C chain | Extracellular Space | other |  |  | **+** |  | 42 | 8 |
| **P01024** | complement component 3 | Extracellular Space | peptidase | IgG |  | **+** | efficacy | 60 | 8 |
| **E9PNW4** | CD59 molecule, complement regulatory protein | Plasma Membrane | other |  |  | **+** |  | 77 | 1 |
| **O43866** | CD5 molecule-like | Plasma Membrane | transmembrane receptor |  |  | **+** |  | 107 | 14 |
| **C9J979** | cleavage and polyadenylation specific factor 3-like | Nucleus | other |  |  | **+** |  | 19 | 1 |
| **D6RAK8** | group-specific component (vitamin D binding protein) | Extracellular Space | transporter |  |  | **+** |  | 53 | 110 |
| **C9JV37** | coagulation factor II (thrombin) | Extracellular Space | peptidase | enoxaparin, desirudin, dabigatran etexilate, Fibrinogen, ximelagatran, antithrombin alfa, aspirin/dabigatran etexilate, dabigatran, argatroban, bivalirudin, lepirudin |  | **+** | diagnosis,  unspecified application | 33 | 1 |
| **P00738** | haptoglobin | Extracellular Space | peptidase |  |  | **+** | diagnosis, efficacy, unspecified application | 58 | 8 |
| **P02790** | hemopexin | Extracellular Space | transporter |  |  | **+** |  | 85 | 12 |
| **P01876** | immunoglobulin heavy constant alpha 1 | Extracellular Space | other |  |  | **+** |  | 81 | 22 |
| **P01857** | immunoglobulin heavy constant gamma 1 (G1m marker) | Extracellular Space | other |  |  | **+** |  | 153 | 17 |
| **P0CG05** | immunoglobulin lambda constant 2 (Kern-Oz- marker) | Extracellular Space | other |  |  | **+** |  | 102 | 11 |
| **P0DJD7** | pepsinogen 5, group I (pepsinogen A) | Extracellular Space | peptidase | sucralfate |  | **+** |  | 32 | 1 |
| **C9JMK5** | phosphoinositide-3-kinase interacting protein 1 | Other | other |  |  | **+** |  | 36 | 1 |
| **P01009-2** | serpin peptidase inhibitor, clade A (alpha-1 antiproteinase, antitrypsin), member 1 | Extracellular Space | other |  |  | **+** | diagnosis, unspecified application | 58 | 9 |
| **H7C4W7** | stabilin 1 | Plasma Membrane | transporter |  |  | **+** |  | 18 | 2 |
| **P02787** | transferrin | Extracellular Space | transporter |  |  | **+** | efficacy, prognosis | 92 | 9 |
| **P01871-2** | immunoglobulin heavy constant mu | Plasma Membrane | transmembrane receptor |  |  | **+** |  | 81 | 11 |
| **Q7L0Y3** | tRNA methyltransferase 10 homolog C (S. cerevisiae) | Cytoplasm | other |  |  | **+** |  | 16 | 2 |
| **P04004** | vitronectin | Extracellular Space | other |  |  | **+** |  | 57 | 9 |
| **Q16853** | amine oxidase, copper containing 3 | Plasma Membrane | enzyme | hydralazine, hydralazine/hydrochlorothiazide/reserpine, hydralazine/hydrochlorothiazide, hydralazine/isosorbide dinitrate |  | **+** |  | 30 | 1 |
| **P08571** | CD14 molecule | Plasma Membrane | transmembrane receptor |  |  | **+** | efficacy | 78 | 8 |
| **E9PNW4** | CD59 molecule, complement regulatory protein | Plasma Membrane | other |  |  | **+** |  | 77 | 10 |
| **J3KRR7** | cylindromatosis (turban tumor syndrome) | Nucleus | transcription regulator |  |  | **+** |  | 28 | 1 |
| **Q8IV36-2** | HID1 domain containing | Plasma Membrane | other |  |  | **+** |  | 18 | 1 |
| **P52272-2** | heterogeneous nuclear ribonucleoprotein M | Nucleus | other |  |  | **+** |  | 41 | 6 |
| **Q8N3X6-2** | ligand dependent nuclear receptor corepressor-like | Nucleus | transcription regulator |  |  | **+** |  | 17 | 1 |
| **P02750** | leucine-rich alpha-2-glycoprotein 1 | Extracellular Space | other |  |  | **+** |  | 74 | 8 |
| **O60487** | myelin protein zero-like 2 | Plasma Membrane | other |  |  | **+** |  | 24 | 1 |
| **Q8NGX3** | olfactory receptor, family 10, subfamily T, member 2 | Plasma Membrane | other |  |  | **+** |  | 20 | 1 |
| **B1AVU8** | prosaposin | Extracellular Space | other |  |  | **+** |  | 29 | 2 |
| **Q5VY30** | retinol binding protein 4, plasma | Extracellular Space | transporter |  |  | **+** | unspecified application | 53 | 4 |
| **G3V3A0** | serpin peptidase inhibitor, clade A (alpha-1 antiproteinase, antitrypsin), member 3 | Extracellular Space | other |  |  | **+** | unspecified application | 62 | 2 |
| **H7C5E6** | solute carrier family 26 (anion exchanger), member 8 | Plasma Membrane | transporter |  |  | **+** |  | 23 | 1 |
| **D6R9C5** | secreted phosphoprotein 1 | Extracellular Space | cytokine |  |  | **+** | diagnosis, efficacy, unspecified application | 23 | 1 |
| **P51809-2** | vesicle-associated membrane protein 7 | Cytoplasm | transporter |  |  | **+** |  | 18 | 1 |
| **Q8N3X6-2** | ligand dependent nuclear receptor corepressor-like | Nucleus | transcription regulator |  |  | **+** |  | 17 | 1 |
| **P02750** | leucine-rich alpha-2-glycoprotein 1 | Extracellular Space | other |  |  | **+** |  | 74 | 8 |
| **O60487** | myelin protein zero-like 2 | Plasma Membrane | other |  |  | **+** |  | 24 | 1 |
| **Q8NGX3** | olfactory receptor, family 10, subfamily T, member 2 | Plasma Membrane | other |  |  | **+** |  | 20 | 1 |
| **B1AVU8** | prosaposin | Extracellular Space | other |  |  | **+** |  | 29 | 2 |
| **Q5VY30** | retinol binding protein 4, plasma | Extracellular Space | transporter |  |  | **+** | unspecified application | 53 | 4 |
| **G3V3A0** | serpin peptidase inhibitor, clade A (alpha-1 antiproteinase, antitrypsin), member 3 | Extracellular Space | other |  |  | **+** | unspecified application | 62 | 2 |
| **H7C5E6** | solute carrier family 26 (anion exchanger), member 8 | Plasma Membrane | transporter |  |  | **+** |  | 23 | 1 |
| **D6R9C5** | secreted phosphoprotein 1 | Extracellular Space | cytokine |  |  | **+** | diagnosis, efficacy, unspecified application | 23 | 1 |
| **P51809-2** | vesicle-associated membrane protein 7 | Cytoplasm | transporter |  |  | **+** |  | 18 | 1 |

**Figure S1.** **Characterisation of serum- and urine-derived EV.** (A, D) Transmission electron microscopy images, (B, E) western blot analysis for exosomal markers (30 μg protein per line), and (C, F) size distribution (one representative patient) of serum- and urine-derived EV, respectively. (G) Average particle number in whole urine compared to urine-derived EV (one representative patient).


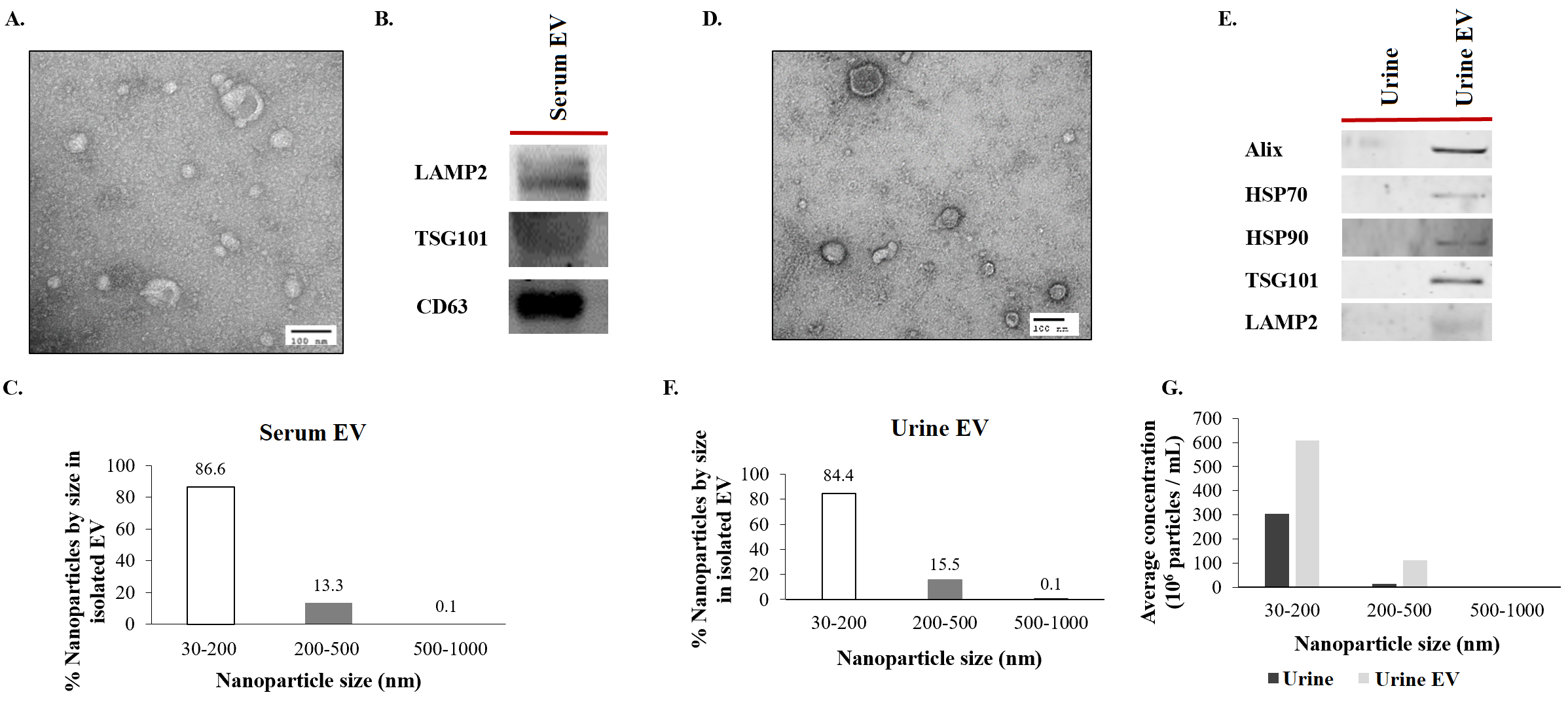


**Figure S2. Coomassie blue staining showing the protein profile of serum and serum-derived EV.** (A) Protein (25 μg) from serum and serum-derived EV of control samples were loaded on SDS-PAGE. (B) Comparison between proteins (15 μg) from serum and serum-derived EV before and after using AlbuminOUT (AO).


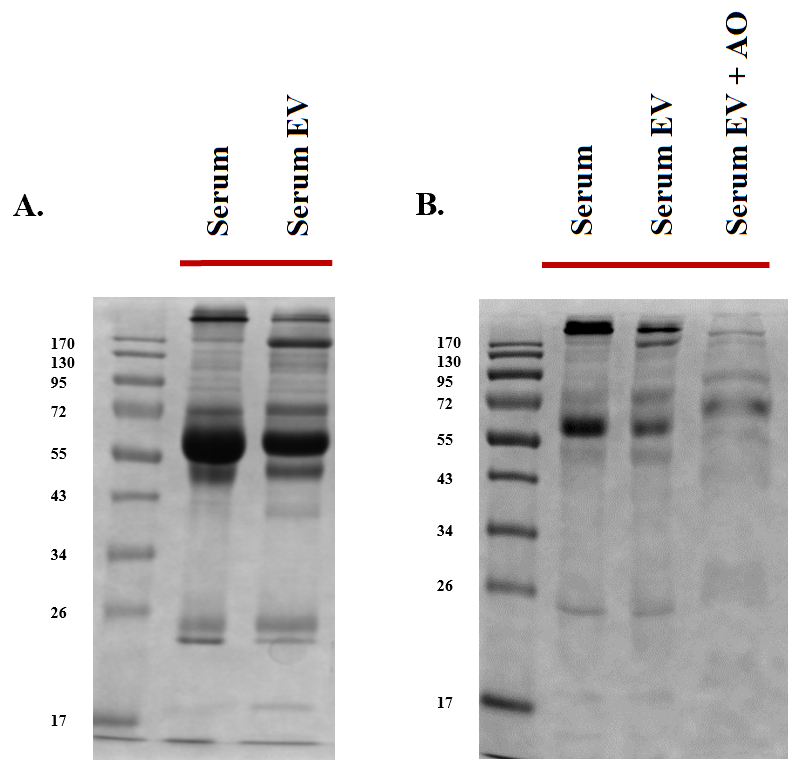


**Figure S3.** Serum-specific EV proteins (not found in urine) identified from six patients who provided matched serum and urine samples.


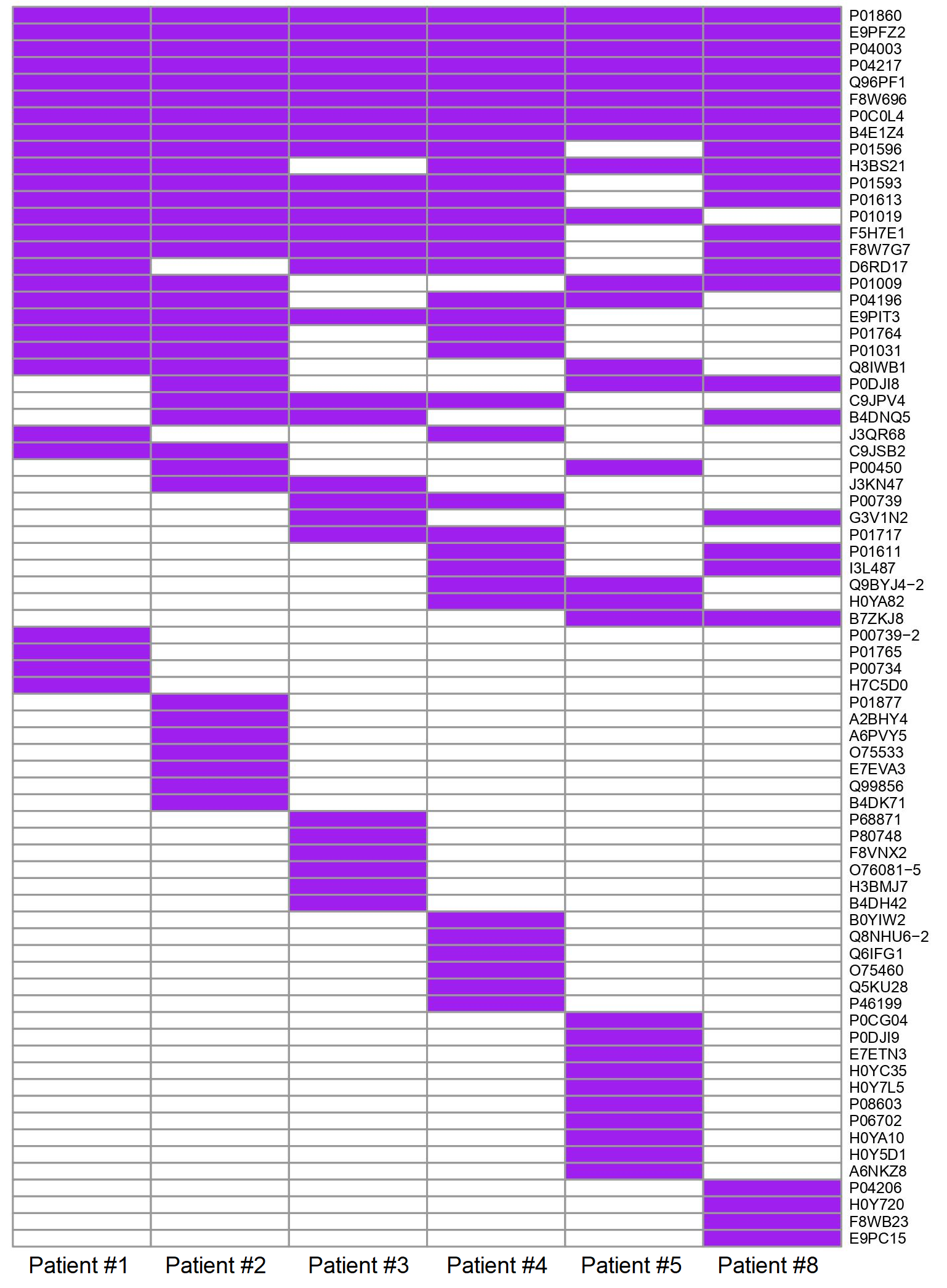


**Figure S4.** Urine-specific EV proteins (not found in serum) identified from six patients who provided matched serum and urine samples.


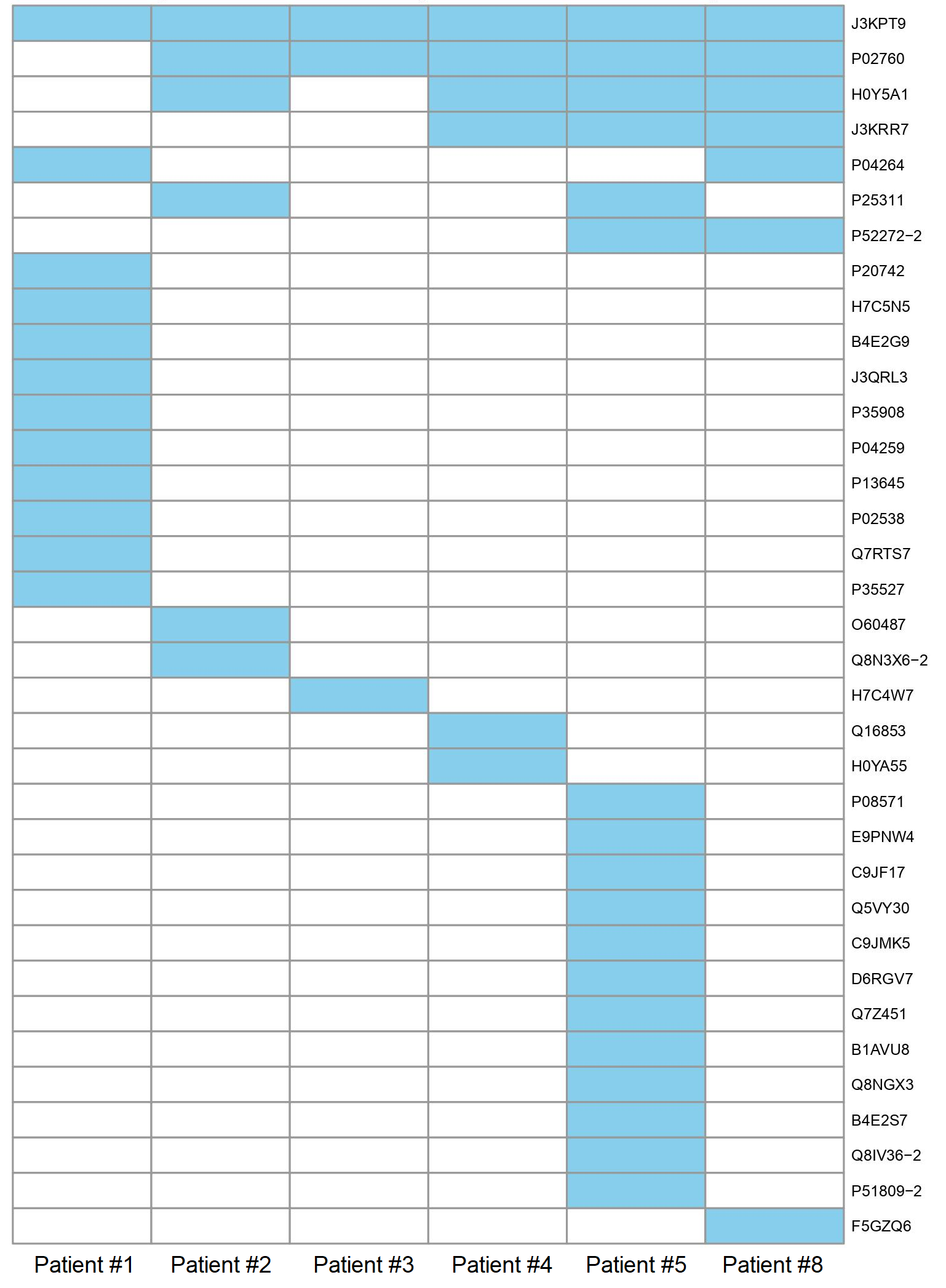

Supplement: Supplementary file 1 — Supplementary file1 (DOCX 4193 KB) [file 432_2022_4391_MOESM1_ESM.docx]
